# Natural Fiber-Reinforced Polylactic Acid Composites: 3D Printing, Life Cycle Assessment, and Applications

**DOI:** 10.3390/polym18172156

**Published:** 2026-09-03

**Authors:** Mpho Phillip Motloung, Khanyisile Sheer Dhlamini, Pauline Ncube, Ntsoaki Joyce Malebo, Mokgaotsa Jonas Mochane

**Affiliations:** 1 Department of Chemistry, College of Science, Engineering and Technology, University of South Africa, Florida Science Campus, Roodepoort, Johannesburg 1710, South Africa; dhlamks@unisa.ac.za (K.S.D.); ncubep@unisa.ac.za (P.N.); 2 Centre for Materials Science, College of Science, Engineering and Technology, University of South Africa, Johannesburg 1710, South Africa; 3 Department of Life Sciences, Central University of Technology, Free State, Private Bag X20539, Bloemfontein 9301, South Africa

**Keywords:** polylactic acid, natural fiber, fused deposition modeling, polymer composites

## Abstract

Natural fiber-reinforced biopolymer composites are an attractive class of materials owing to their renewability, sustainability, and eco-friendliness. Meanwhile, fused deposition modeling (FDM) is transforming the manufacturing industry and gaining popularity for fabricating intricate and customized geometries that are difficult to produce with traditional techniques. Recently, FDM printing of natural fiber-reinforced PLA composites (NFRPCs) has emerged as a promising approach for developing sustainable green composites with potential application across diverse fields. This review comprehensively examines recent advances in the development of FDM-printed NFRPCs, with particular emphasis on mechanical and tribological properties. In addition, the review examines life cycle assessment (LCA) studies to evaluate the environmental sustainability of FDM-printed NFRPCs and identifies important gaps that require further investigation. Challenges associated with the processing of NFRPCs, including printability, interfacial adhesion, and optimal printing parameters, are also highlighted. Finally, future research directions are proposed to facilitate the development of high-performance, printable, and environmentally sustainable NFRPCs.

## 1. Introduction

In the modern world, as we seek materials that minimize adverse impacts on ecosystems and society, natural fibers have come to the forefront, largely because of their unique properties. Natural fibers are renewable, sustainable, non-toxic, corrosion-resistant, and eco-friendly materials. These materials have generated tremendous interest as reinforcements in polymers, cements, and ceramics, mainly because they are abundant and available at low-to-zero costs [1,2,3,4,5]. Natural fibers have low density, offer an alternative to synthetic fibers, and have lower environmental impact. For example, one study reported a life cycle assessment (LCA) of flax and glass fiber production. Across the evaluated categories, flax fiber production showed 47%, 73%, and 58% reductions in global warming potential (GWP), marine ecotoxicity potential, and ozone depletion potential (ODP), respectively [6]. These findings underscore the advantage of natural fibers over synthetic glass fibers, particularly during their production. Similarly, sisal fiber production shows much lower greenhouse gas (GHG) emissions and non-renewable energy use than glass fiber [7].

Natural fiber-reinforced polymer composites (NFRCs) are attractive materials that can be used in diverse applications, including automotive, aerospace, construction, medicine, armor, and sports equipment, among others [2,8]. In this context, biodegradable polymer composites reinforced with natural fibers are particularly intriguing because of their renewability, sustainability, and environmental friendliness [9]. Polylactic acid (PLA) composites reinforced with natural fibers have emerged as a new class of materials with immense interest, largely due to the aforementioned merits. PLA and natural fibers are derived from renewable resources and are biodegradable and biocompostable [10]. PLA is a biopolymer produced in large quantities compared with other synthetic biopolymers, and it has the potential to replace most conventional polymers across various applications. This polymer exhibits desirable properties that make it attractive in applications, such as packaging, automotive, medical devices, and mulch films. The incorporation of natural fibers into PLA represents a feasible strategy to minimize its costs and further widens its application scope [10,11]. Furthermore, the incorporation of natural fibers offers additional benefits over inorganic fillers, such as renewability, biodegradability, low density, and sustainability [12].

Numerous studies have been reported on the development of natural fiber-reinforced PLA composites (NFRPCs) [13,14,15,16]. These studies investigated several key characteristics of these composites, such as structure–morphology relationships, which translate to mechanical performance. Due to their hydrophilic nature, natural fibers often exhibit poor adhesion with the PLA matrix. Therefore, they are usually chemically modified to enhance fiber–matrix adhesion. Several studies have investigated the effects of chemical fiber treatments on the adhesion, morphology, and properties of NRFPCs [17,18,19]. Flammability of NFRPCs has also been investigated. Wagner et al. [20] evaluated flammability properties of PLA reinforced with hemp, flax, and sisal fibers. PLA-based hybrid composites containing natural fibers have also generated substantial interest because they often exhibit enhanced properties compared to single-fiber composites. Hybrid composites combining different natural fibers or natural fiber with nanofillers exhibit enhanced properties compared to single-fiber-reinforced PLA composites. Hybrid PLA composites containing bamboo fiber (10 wt. %) and montmorillonite clay (4%) could achieve 26.1% and 40.8% increases in tensile strength and modulus, respectively [12]. In another study [21], it was shown that incorporating sisal and coir fibers as single fibers in PLA could enhance tensile strength and impact resistance, respectively. However, dual fibers improved both impact resistance and tensile strength, highlighting the significance of hybridization. Recently, the development of NFRPCs through advanced technologies such as additive manufacturing (AM) has grown substantially, largely because it enables the production of intricate and customized structures.

AM, or three-dimensional (3D) printing of thermoplastics, has grown significantly in recent years, mainly because it can produce intricate geometries, enable rapid prototyping, and reduce material waste [22,23]. Fused deposition modeling (FDM) is an AM technology that has attracted enormous attention for printing thermoplastics, such as PLA. FDM printing of PLA has grown substantially over the years due to good printability, minimal warping, and cracking [24,25]. Due to its good printability, FDM printing of PLA has translated to printing of its composite-based materials as a viable way to expand its processing and utilization in diverse application fields. FDM printing of NFRPCs has garnered substantial attention, aiming to advance their use and broaden their application scope. As with any NFRCs, several key characteristics influence the material properties of FDM-printed NFRPCs. Generally, the interfacial adhesion between PLA and natural fibers, fiber size, and loadings affect the resulting properties of these materials. Chemical treatments of the natural fibers and the addition of compatibilizers also provide an alternative route to improve interfacial interactions and load-bearing properties. Moreover, printing parameters, such as infill properties and raster angles, significantly affect the properties of FDM-printed NFRPCs. Recent reviews have critically explored various aspects related to filament preparation methods, processing, structure–property relationships, and biodegradation in FDM-printed NFRPCs [26,27,28]. This review critically examines how material characteristics and printing conditions affect the mechanical and tribological properties of FDM-printed NFRPCs. Furthermore, the review extends beyond the conventional structure–property assessment in NFRPCs by considering the environmental impacts through LCA, thereby highlighting potential trade-offs between material processing, performance, and sustainability. The review also highlights challenges and limitations when fabricating these materials and provides recommendations and future research directions when designing 3D-printed NFRPCs.

## 2. Natural Fibers

**Figure 1:**
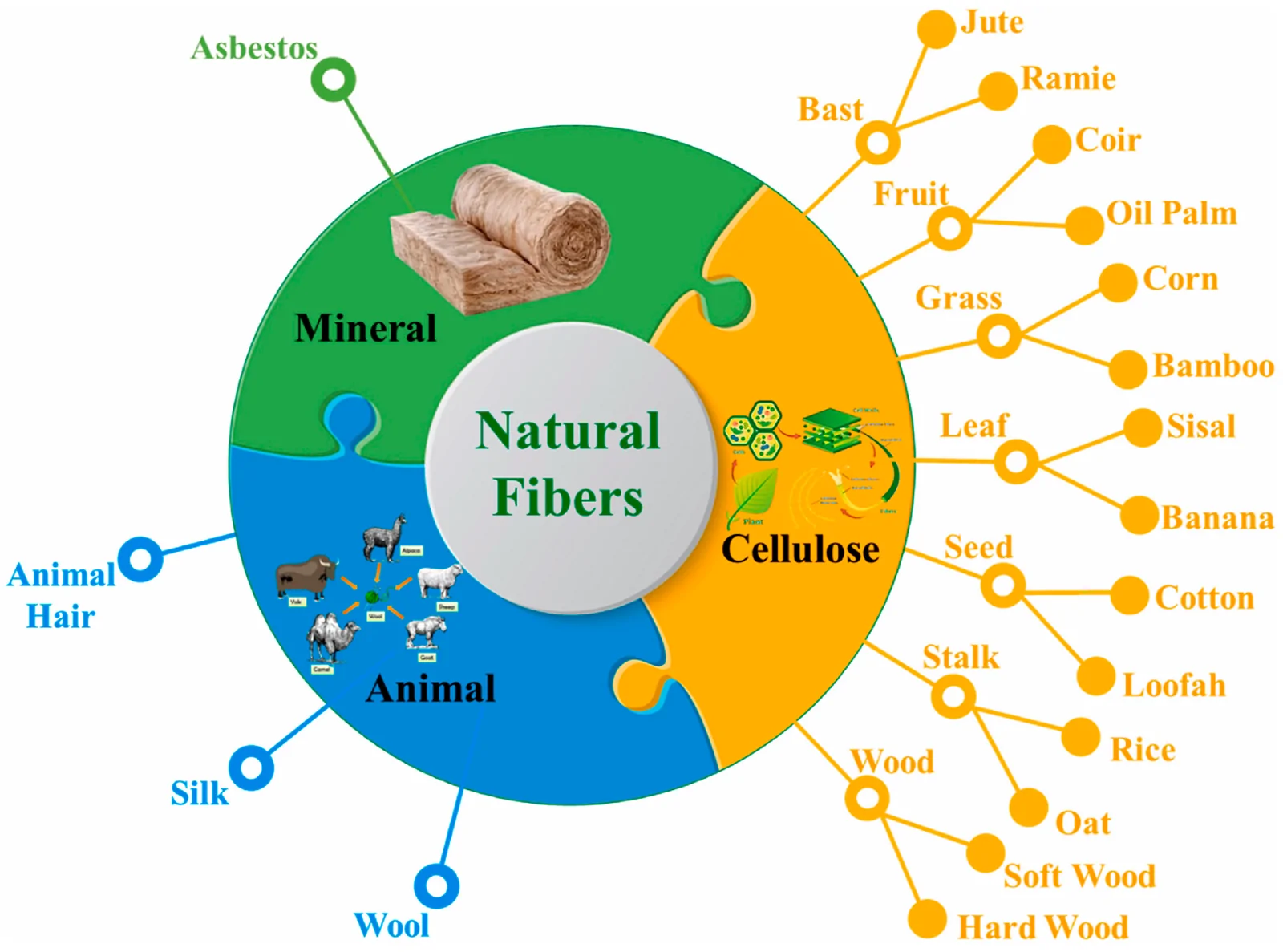
Classification of natural fibers. Reproduced from [3]. Elsevier, Creative Commons CC-BY.

Synthetic fibers such as carbon, aramid, and glass fiber have been extensively used in applications such as construction, automobiles, aeronautics, and many others. Despite their widespread use, these fibers have disadvantages that limit their broader utilization. For instance, glass fiber exhibits excellent mechanical properties; however, it is associated with adverse effects on the environment and human health, including risks of lung cancer and skin allergies. Rapisarda et al. [29] evaluated the cytotoxicity, genotoxicity, and oxidative stress induced by exposure of human alveolar epithelial cells (A549 cells) to glass fibers. The in vitro studies demonstrated that exposure to glass fibers reduced cell viability and increased DNA damage and oxidative stress in A549 cells. Similarly, studies investigating the cytotoxicity and oxidative effects of man-made vitreous fibers (MMVFs) in a human mesothelial cell line (MeT-5A) reported direct DNA damage and loss of microvilli, confirming the potential cytotoxicity, genotoxic, and oxidative effects of various MMVFs, including rock wool, refractory ceramic fibers, and glass wool [30]. Hence, there is a growing need to replace synthetic fibers with non-toxic and environmentally benign alternatives [2,3,10]. Natural fibers, obtained from renewable resources, serve as an alternative to synthetic fibers. Figure 1 depicts the various sources of natural fibers, showing that most natural fibers come from plants. Natural fibers derived from plants (e.g., leaves, stems, grass, fruits, bast, etc.) are mostly used compared to animal and mineral fibers. Mineral fibers such as asbestos have been widely used in buildings; however, many countries have banned them because of health concerns, such as cancer, associated with them [2,3,8]. Therefore, there is a significant shift toward environmentally friendly, renewable plant-based fibers.

LCA is an important tool for evaluating the environmental sustainability of materials throughout their entire life cycle, from their production to end of life. This tool is imperative because it helps identify more sustainable methods for extraction, processing, and utilization of natural fibers [31,32,33]. LCA allows optimization of processing costs from cultivation for fiber extraction, while also identifying environmental hotspots that require improvements. There are studies that have comparatively assessed the environmental impacts of various natural fibers used in textile applications. Cotton is widely used in textiles and has been compared with other natural fibers, including hemp, kenaf, and jute [34,35]. These studies demonstrated that cotton production generally has the highest environmental impacts compared to alternative natural fibers, particularly in terms of eutrophication potential (EP), GWP, acidification, land use, and mineral resource use. For example, cotton farming was associated with an EP of approximately 60 kg N eq, compared to hemp fiber with only 3 kg N eq. The highest EP largely stemmed from the extensive use of agrochemicals and fertilizers in cotton farming [34]. Similarly, traditional cotton fiber production showed the highest GWP of 2446 kg CO_2_-eq, compared with 294 and 360 kg CO_2_-eq for jute and kenaf, respectively [35]. However, organic cotton production significantly reduces these impacts, highlighting the importance of LCA in identifying and refining production methods and processes to reduce the environmental footprint of natural fibers. Plant-based fibers have gained enormous attention among other natural fibers. One study evaluated the environmental impacts of various textile fibers used, including organic cotton, jute, flax, silk, and polyester, using a cradle-to-gate LCA approach that assessed multiple stages of the production process [36]. Across the natural fibers evaluated, silk had the highest environmental impact at the agricultural stage across all assessed categories, including fossil resource scarcity, freshwater ecotoxicity and eutrophication, GWP, human carcinogenic toxicity, land use, water consumption, and marine ecotoxicity. Silk production requires the cultivation of mulberry trees, which serve as a food source for silkworms. After the silkworms’ cocoons are collected, the cocoons are boiled to extract the fibers. These findings support growing interest in plant-based natural fibers over animal-derived fibers, such as silk.

**Figure 2:**
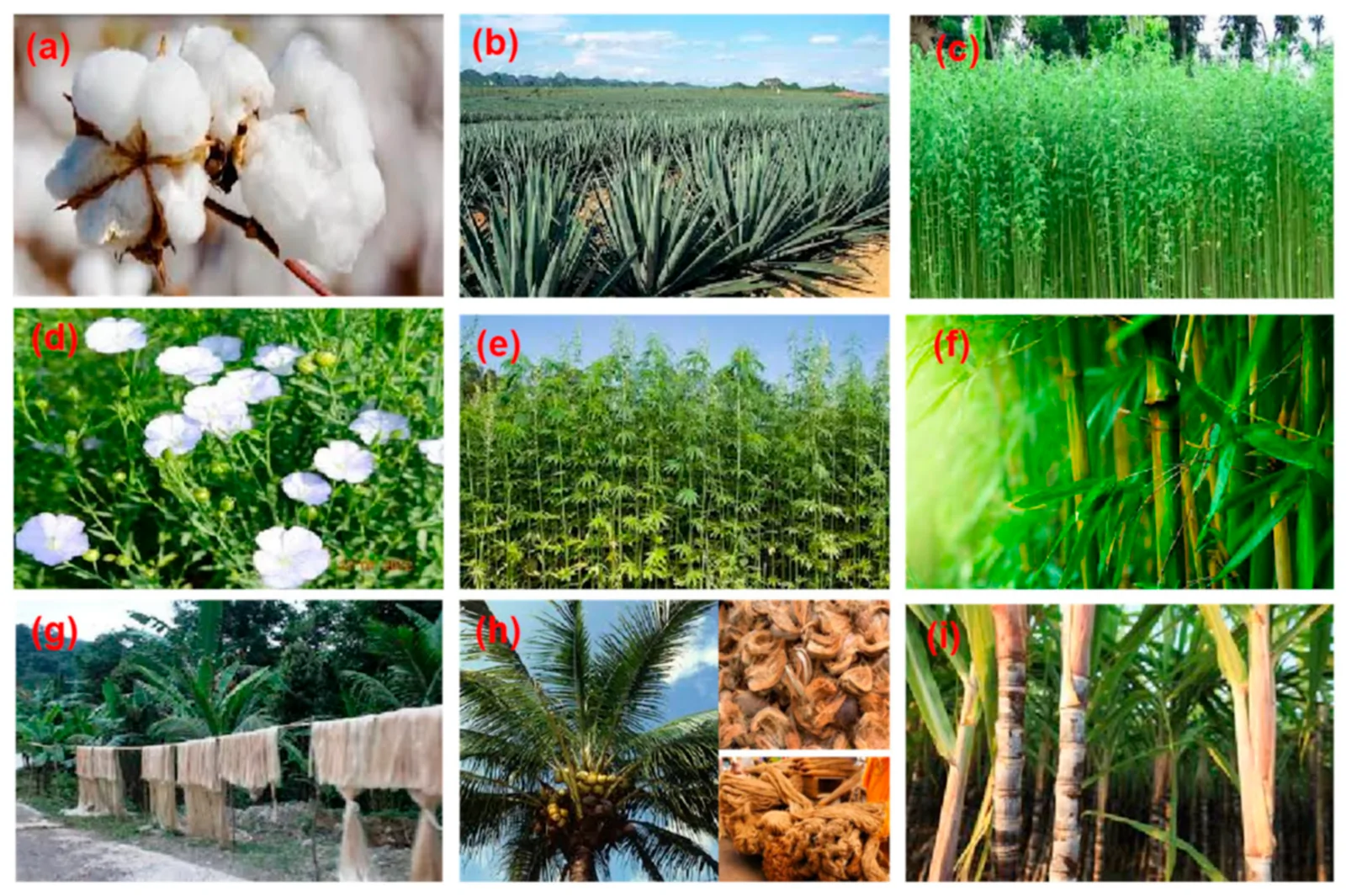
Common natural fiber plants: (**a**) cotton; (**b**) sisal; (**c**) jute; (**d**) flax; (**e**) hemp; (**f**) bamboo; (**g**) banana; (**h**) coir; (**i**) sugar cane. Reproduced from [40]. MDPI, open access.

Plant-based fibers are abundant and available in large quantities. Figure 2 illustrates some of the common plant fiber sources. Fibers such as rice husk (RH), wood fiber, bamboo, sugarcane bagasse, and jute are among the most produced fibers worldwide, with countries such as Brazil, China, and India being at the forefront of their production [3]. Plant fibers have been utilized by humans for thousands of years and are among the first materials to be used for textiles and other useful applications, with hemp being considered one of the earliest cultivated fibers, used around 2800 BCE [37]. However, the use of other plant fibers such as cotton, sisal, hemp, coir, and bamboo has grown substantially over the past decades, particularly in producing paper, ropes, mats, and other industrial fabrics. This has been largely driven by the rising environmental risks and consumer health concerns associated with petroleum-based resources. Due to their low cost, abundance, versatility, biodegradability, recyclability, and low carbon footprint, plant fibers have emerged as sustainable alternatives to synthetic/petroleum-based materials [37]. Additional advantages, including low density, good fiber aspect ratio, and comparatively high tensile strength and flexural moduli, have expanded their use in cutting-edge industries such as geotextiles, polymer composite reinforcements, and other advanced engineering applications. However, plant-based fibers have numerous challenges that limit their applications. Generally, plant-based natural fibers exhibit relatively low thermal stability and high flammability, which limits their utilization in high-temperature applications. In addition, their hygroscopic nature and high moisture absorption capacity make them susceptible to microbial attack, accelerating degradation and compromising long-term durability. Hygroscopic aging, therefore, presents a significant challenge, as repeated moisture absorption can adversely affect the mechanical and dimensional stability of natural fibers. Moisture uptake causes fiber swelling, which can induce dimensional changes and weaken the fiber structure, ultimately reducing its mechanical performance [1,38,39].

Plant fibers are classified as bast, leaf, stalk, and seed or fruit according to the part of the plant from which they are derived [3] (Figure 1). Bast is made up of a wood core surrounded by a stem consisting of several fiber bundles with individual fiber cells or filaments within them. Examples include ramie, kenaf, and hemp. Leaf fibers are usually coarser than bast fibers and include sisal, banana, and pineapple. Cotton and coir are the most common seed and fruit fibers, respectively. Stalk fibers include oat, maize, and wheat straws. Other sources of lignocellulosic fibers include agricultural waste, such as bagasse from sugar mills, sunflower seed hulls from oil processing, and rice hulls from rice production [4,41].

### 2.1. Chemical Composition and Structure of Plant-Based Fibers

**Figure 3:**
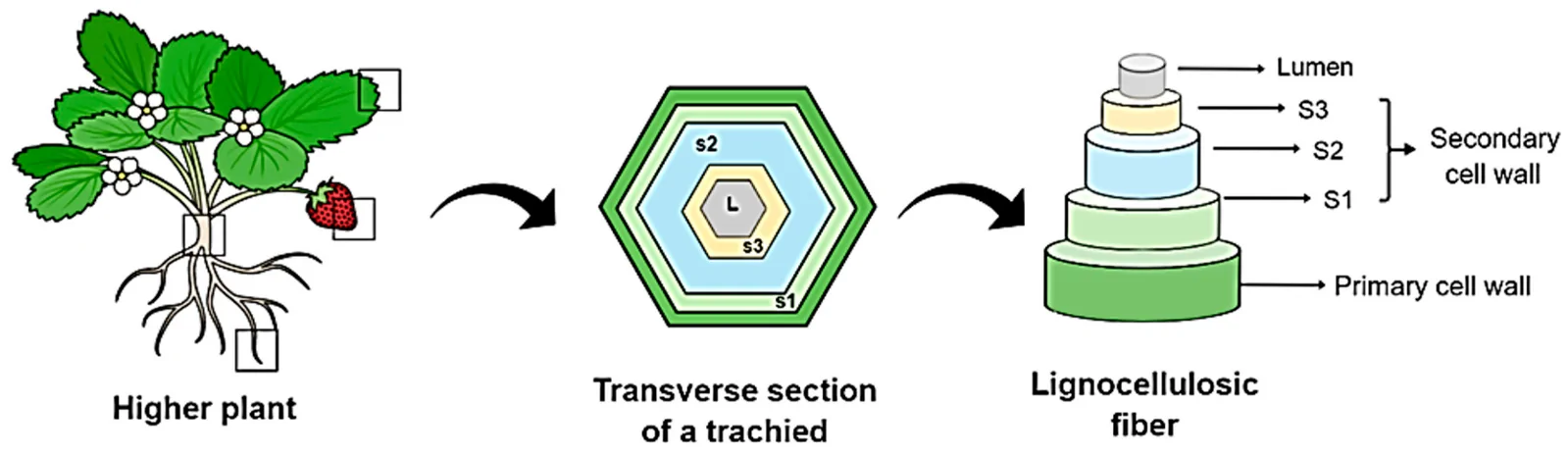
Typical structure of plant fiber. Reproduced from [44]. MDPI, open access.

Plant fibers are lignocellulosic materials that are primarily composed of cellulose, hemicellulose, and lignin, with minor amounts of pectin, ash, and waxes, as well as moisture [42]. Table 1 summarizes the chemical compositions of various plant fibers. The precise composition and structure vary with plant species, maturity, climate, region of growth, and extraction technique. Cellulose exhibits a semi-crystalline structure responsible for the strength, stiffness, and structural stability of the fiber. The amorphous hemicellulose functions as a cementing agent that stabilizes the fiber structure by forming hydrogen bonds with cellulose fibrils [9,43]. Additionally, the hydrophilic hydroxyl groups (–OH) in cellulose and hemicellulose facilitate moisture absorption. While lignin is a complex aromatic polymer that provides rigidity and protection to the fiber cell wall, waxes constitute surface impurities that affect the wettability and adhesion of the fiber matrix [4,43,44]. Figure 3 illustrates the typical structure of plant fibers. Plant fiber cell walls are composed of a primary wall with a loosely packed network of cellulose microfibrils and a secondary wall made up of three distinct layers: S1 (outer layer), S2 (middle layer), and S3 (inner layer). The S2 layer is the thickest and plays a key role in determining mechanical properties. The ratio of cellulose to lignin/hemicellulose and the orientation (microfibrillar angle) of cellulose differ between fibers [4,43]. Fiber strength and stiffness are primarily influenced by cellulose content, microfibrillar angle, and degree of polymerization. Generally, fibers with higher cellulose content, smaller microfibrillar angles, and a greater degree of polymerization exhibit better mechanical properties [9,43]. Pectin is also present in the fibers, and it provides flexibility to the fibers, whereas waxes form an outer layer on the fibers. Furthermore, the fibers also contain inorganic ash components, which are responsible for the abrasiveness of the fibers [9].

### 2.2. Extraction Methods

To produce fiber-based products, it is essential to first separate natural fibers from their source. Various extraction methods are used, with retting being the most common. Retting uses microbes such as bacteria and fungi to break down the pectic polysaccharides that bind fiber bundles to surrounding plant tissue. Other methods include chemical treatments or mechanical processes. Importantly, fiber type determines the choice of extraction method, which significantly affects chemical composition, structure, fiber yield, and overall quality. Table 2 summarizes common natural plant fiber extraction methods [1].

Table 3 summarizes different extraction methods employed to extract various plant-based natural fibers. Different extraction methods affect the chemical composition of the resulting fibers. The chemical extraction method has been shown to produce fibers with different compositions. In some cases, the cellulose content in the fibers decreased significantly with increasing the concentration of alkali solution in chemical extraction methods [47,48]. Kacem et al. [47] employed various methods to extract Yucca fibers. In these methods, it was observed that increasing the alkali concentration from 3 to 10% decreased the cellulose content from 73.95 to 65.85%, respectively. Higher concentration degrades cellulose in fibers, suggesting that optimum concentrations are required to attain fibers with high cellulose content. The amount of cellulose in fibers can be correlated with the mechanical properties of the fibers, where fibers with high cellulose content have been reported to exhibit improved mechanical properties. For instance, in a study by Kacem et al. [47], it was observed that Yucca fibers extracted with 3% and 10% sodium hydroxide exhibited cellulose contents of 73.95% and 65.85%, and corresponding tensile strengths of 456.23 MPa and 235.53 MPa, respectively. Overall, different extraction methods affect the composition and the properties of natural fibers.

## 3. Polylactic Acid: Synthesis and Properties

**Figure 4:**
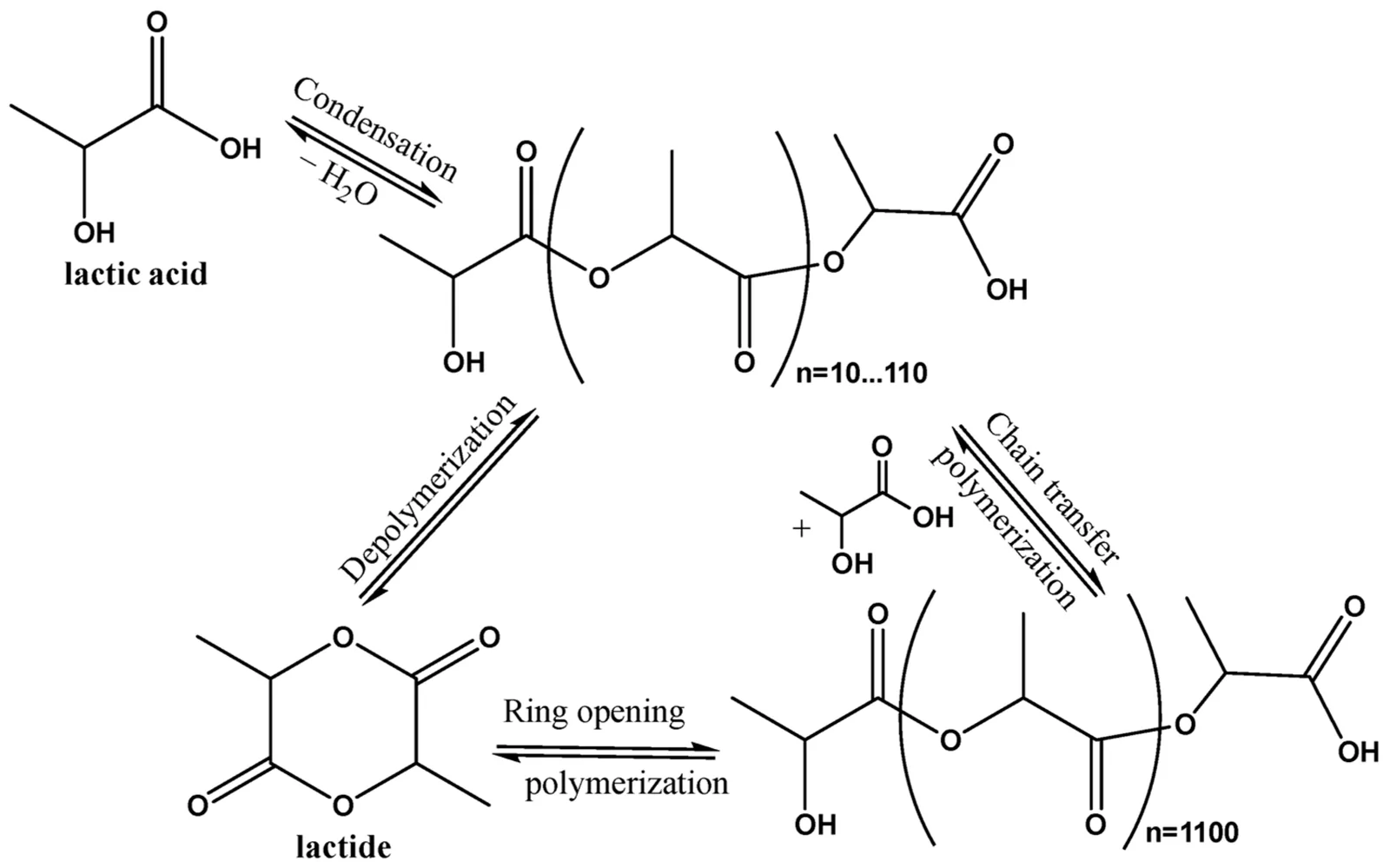
Synthesis of PLA from lactic acid and lactide. Reproduced from [57]. MDPI, open access.

PLA is an aliphatic polyester that is bio-based, as it is derived from renewable resources such as corn, sugarcane, etc. Generally, PLA is synthesized from chiral lactic acid that is obtained by either chemical methods or microbial fermentation of sugars from these renewable resources. Lactic acid monomer exists in two enantiomeric forms (L-lactic acid and D-lactic acid) or a racemic mixture of the two [52,53]. Synthesis of PLA is essentially through polycondensation of lactic acid, though this method remains less favored due to the release of water as a byproduct that adversely affects the molecular weight and overall physicochemical properties of PLA. Lactide is a cyclic monomer derived from lactic acid through the depolymerization of low-molecular-weight PLA. Lactide can exist as a homochiral (L, L- and D, D-) molecule or a heterochiral molecule (L, D-), depending on the enantiomeric form of lactic acid [52]. Figure 4 depicts a typical synthesis route for producing PLA from lactic acid and lactide. Synthesis of PLA from lactide is by ring-opening polymerization. This method is industrially favored as it yields PLA with a high molecular weight. Both lactic acid and lactide exist in different stereoisomers; therefore, PLA also exists in different stereoisomers (e.g., PDLA, PLLA, and PDLLA). PLLA and PDLA are semi-crystalline forms of PLA, while PDLLA is an amorphous polymer [52,53,54,55]. PDLLA may be particularly suitable for FDM printing because its amorphous structure reduces crystallization-induced shrinkage, which can occur in semi-crystalline PLA stereoisomers such as PLLA during cooling. In contrast, PDLA crystallizes more slowly, which may reduce crystallization-induced shrinkage and warping during printing. A low crystallization rate is crucial because it keeps PLA highly amorphous, which promotes layer adhesion [56].

PLA is processable by a wide range of techniques, including extrusion, blow molding, injection molding, fiber spinning, and others. It exhibits high clarity and gloss, with balanced mechanical properties. As a result, PLA is used in applications such as bottles and single-use items, such as cutlery and cups [58]. However, it is a brittle, rigid polymer with poor toughness, which limits its use in other applications. The addition of plasticizers [59], blending with ductile polymers [60,61], or co-polymerization with other monomers [62] improves PLA toughness and impact properties. 3D printing of PLA has generated immense attention due to the ease of printing of PLA and minimal warping and cracking [24,25]. Usually, PLA is printed at low temperatures (~180–210 °C) and bed temperatures in the range of 50–60 °C, which is not necessarily required for materials such as PLA [63]. However, the nozzle or extrusion temperatures should be determined based on the specific commercial grade and manufacturer’s recommendations, followed by optimization according to the composite formulation. The printing temperatures for PLA are fairly low considering the printing temperatures required to print other polymers such as acrylonitrile butadiene styrene (ABS), polyethylene terephthalate glycol, and polycarbonate. PLA does not form cracks like other polymers used in 3D printing, such as ABS. It is also desired for its high print quality and excellent surface finish, making it essential for aesthetic applications.

## 4. Natural Fiber-Reinforced PLA Composites (NFRPCs)

The use of natural fibers as reinforcing agents has increased significantly over the years, largely because of the interesting key features they exhibit. Researchers have widely reported the development of natural fiber-reinforced PLA composites. Incorporating various natural fibers into PLA improves mechanical properties because the fibers enable the matrix to distribute and absorb applied stress. Moreover, fibers increase the composite’s impact resistance by absorbing and distributing impact energy. Owing to their environmental friendliness, NFRPCs are potentially an alternative to synthetic fiber-reinforced composites, particularly in developing sustainable materials for advanced applications. Generally, the properties and performance of NFRPCs largely depend on interfacial adhesion between PLA and the fibers [10,21,64]. Natural fibers are hydrophilic, and their interaction with PLA is often weak, resulting in poor matrix–fiber adhesion and poor wetting of the fibers by the matrix [2]. Generally, NFRPCs face several challenges that can affect their long-term durability and performance. Although these materials offer significant environmental benefits, their susceptibility to environmental degradation can limit their long-term application. In particular, their mechanical properties can deteriorate when exposed to humid environments, ultraviolet (UV) radiation, chemicals, and temperature fluctuations. These environmental stresses can induce fiber swelling, matrix cracking, and fiber–matrix debonding, thereby weakening the structural integrity of the composites. Moisture absorption is particularly important because the hydrophilic nature of natural fibers promotes water uptake, which can cause fiber swelling, dimensional changes, interfacial degradation, and, under suitable conditions, hydrolytic and biological degradation. In addition, PLA is susceptible to moisture-induced hydrolytic degradation, which can reduce molecular weight and deteriorate its mechanical performance [38,39]. Consequently, simultaneous degradation of the natural fibers, polymer matrix, and fiber–matrix interface can significantly affect the structural and bulk properties of NFRPCs under prolonged environmental exposure. Wang et al. [65] investigated the effect of hygrothermal aging on the mechanical properties of PLA composites reinforced with flax fibers. They observed a significant reduction in tensile strength and stiffness with increasing aging duration up to 42 days. Similarly, Jiang et al. [66] investigated the influence of hydrothermal aging on PLA/jute composites by exposing the materials to distilled water at different temperatures (23, 37.8, and 60 °C). Their findings demonstrated that aging temperature strongly influenced the degradation of the composite properties. The mechanical properties and molecular characteristics deteriorated more rapidly at 60 °C than at lower temperatures, where degradation occurred more gradually. Interestingly, the unaged samples exhibited good interfacial compatibility, whereas samples aged at 60 °C for 28 days developed cracks and exhibited weakened fiber–matrix interfacial bonding, indicating interfacial debonding. Therefore, understanding the aging mechanisms of NFRPCs is crucial for improving their durability, reliability, and service life, particularly in applications involving prolonged exposure to adverse environmental conditions. To overcome this challenge, natural fibers are chemically treated through various methods, such as alkali treatment and silanization, to improve their compatibility with the matrix. Chemically treated fibers interact more strongly with PLA, improving properties such as load-bearing capacity and impact resistance. Although chemical treatments effectively enhance compatibility, they are less favored because of their harshness and may increase overall production costs [67]. Hence, alternative methods with lower environmental impact are needed to improve compatibility between plant-based natural fibers and PLA. Yadav, Ritu, and Singh [67] comparatively investigated methods for modifying banana and bagasse fibers through natural and chemical treatments. The chemical treatment used a typical alkali treatment with a 5% sodium hydroxide solution. The natural treatment involved treating the fibers with *Sapindus mukorossi*, *Azadirachta*, and citric acid. The LCA analysis demonstrated the highest environmental impact when using chemical treatment compared to the natural treatment method. However, the natural method had a high impact on land use, eutrophication, marine and terrestrial acidification, and ecotoxicity because cultivating *Sapindus mukorossi* and *Azadirachta* requires fertilizers. Another study compared the environmental impact of treating kenaf fibers with bio-based tartaric acid (5%) and sodium hydroxide solutions (5%) [68]. The LCA assessment used ReCiPE 2016 Endpoint (E) V1.03/World (2010) E/A. Alkali treatment showed the highest environmental impact compared with tartaric acid treatment across all assessed categories, including global warming, ozone depletion, eutrophication, acidification, and human non- and carcinogenic toxicity. Conducting LCA of different fiber treatments could improve environmental sustainability and potentially reduce the costs associated with modification methods.

The incorporation of compatibilizers such as PLA-grafted maleic anhydride (PLA-g-MA) presents another effective route to promote interfacial adhesion and wetting of the fibers by the PLA matrix [69,70]. Reactive extrusion also allows in situ compatibilization of the polymer matrix and incorporated natural fiber [71]. Future research should also focus on LCA of these methods, compared with chemical fiber treatments. Generally, the mechanical properties of natural fiber-reinforced polymer composites depend on interfacial properties; therefore, improving these properties is necessary to develop composites with enhanced load-bearing capacity.

NFRPCs are a sustainable, eco-friendly, and renewable class of materials with potential for use in diverse application fields. Nevertheless, NFRPCs have drawbacks that restrict their widespread use. These materials are highly flammable and tend to absorb significant amounts of moisture, which eventually affects their properties due to hygroscopic aging. Improving these drawbacks is imperative, as it widens their application scope [71,72,73,74,75,76,77]. Hybridization, which refers to combining two or more fibers or a fiber with another filler (e.g., a nanomaterial), has generated immense interest in producing NFRCs with improved properties compared to single-fiber-reinforced polymer composites. Hybridization broadens the application of NFRPCs [2]. For instance, the incorporation of carbon nanotubes into PLA/bamboo fiber composites improves the electrical conductivity of the composites and the electromagnetic interference (EMI) shielding effectiveness [78]. In this study, the application scope of PLA/bamboo fiber composites can now be extended to applications requiring EMI shielding through hybridization with conductive carbon nanomaterials. In another study [79], it was shown that 9,10-dihydro-9-oxa-10-phosphaphenanthrene-10-oxide (DOPO)-functionalized carbon nanotubes can effectively improve the tensile strength and flammability properties of PLA/ramie fiber biocomposites. These studies prove the significance of hybridization in NFRPCs. Moreover, hybridization provides opportunities for cost optimization, weight reduction, and broadening the range of potential applications of natural fiber-reinforced composites. However, hybridization also presents several drawbacks, including the need for uniform dispersion of multiple reinforcing phases, potential incompatibility between the constituent materials and the polymer matrix, and increased processing complexity, which may adversely affect the overall cost–performance balance. In addition, hybrid composites may develop internal stresses due to differences in the coefficients of thermal expansion, degradation behavior, and moisture absorption of the incorporated fibers and other reinforcing fillers [80,81].

NFRPCs hold great potential for utilization in automotive, electronics, textiles, and electronic packaging. Patents play a pivotal role in advancing technological innovation by helping to identify emerging trends, industrial priorities, and opportunities for future research. In the area of PLA/natural fiber composites, several patents have been reported and are summarized in Table 4. These patents primarily focus on enhancing biodegradability, heat resistance, and mechanical performance of PLA/natural fiber composites. Moreover, most patents address key challenges in processing PLA, such as degradation during manufacturing. These patents include strategies to overcome these limitations, such as eliminating solvent-based routes, reducing residence time during thermal processing, improving natural fiber dispersion within the PLA matrix, and optimizing extrusion and molding temperatures to minimize thermal degradation. For instance, a patent by Ineos Styrolution Group titled “Polylactic acid composites with natural fibers” introduced a solvent-free process of preparing PLA/natural fiber composites. The method enabled improved dispersion of natural fibers in PLA, reduced processing time, and improved mechanical properties of the resulting composite. These patents provide insight into industrial innovation, and they can facilitate technology transfer and help identify emerging technologies and trends. However, NFRCs in general, and NFRPCs in particular, face significant challenges to widespread industrial adoption because standardized testing protocols for evaluating long-term durability and performance under diverse environmental conditions are lacking. Arunprasath et al. [38] proposed standards for evaluating the durability of these composites under different environmental exposures. Implementing such standardized protocols could establish consistent durability benchmarks, facilitate material certification, and improve the reliability and long-term performance of NFRCs.

The growing interest in NFRPCs has stimulated research into advanced manufacturing techniques for processing these materials. AM has emerged as a promising approach for fabricating NFRPCs, largely because it offers design flexibility and can produce intricate shapes compared to traditional techniques such as injection and compression molding. In particular, FDM-based printing has garnered considerable attention because of its versatility in fabricating intricate shapes and customization with relatively low material waste. This technology has transformed NFRPC fabrication by enabling complex geometries that cannot be achieved with traditional processing techniques. However, incorporating natural fibers introduces additional drawbacks related to printability, fiber dispersion, interfacial adhesion, nozzle clogging, and the anisotropic nature of FDM-printed structures. These factors can significantly affect the mechanical properties and the performance of NFRPCs.

## 5. Additive Manufacturing

3D printing is a rapidly growing technology that has revolutionized the manufacturing industry. This technology has advanced the manufacturing sector by allowing the development of complex 3D geometries that are difficult to attain with traditional techniques. AM is versatile and is favored due to its customization capabilities and prototyping with reduced material waste [23,86]. Additionally, the interest in AM is largely driven by its capacity to develop lightweight materials. With this technology, a diverse range of materials, including metals, ceramics, and polymers, can be processed into different geometries. Generally, complex geometries are built from digital models developed with computer-aided design (CAD) software [87]. AM manufacturing technologies vary across the diverse materials they can print. Common AM technologies include stereolithography, digital light processing, selective laser sintering, direct ink writing, and FDM [88]. Among these technologies, FDM has gained substantial interest in printing thermoplastic filaments through a heated nozzle. In FDM, a thermoplastic filament is heated in an extruder head and then printed layer by layer to build a desired 3D object from models prepared with CAD software. Thermoplastics such as PLA and ABS are widely printed with FDM. This technology has several advantages, including cost-effectiveness, low maintenance, reproducibility, and rapid prototyping [87,88]. Most recently, FDM printing of natural fiber-reinforced composites is gaining substantial momentum.

### 5.1. FDM Printing of NFRPCs

**Figure 5:**
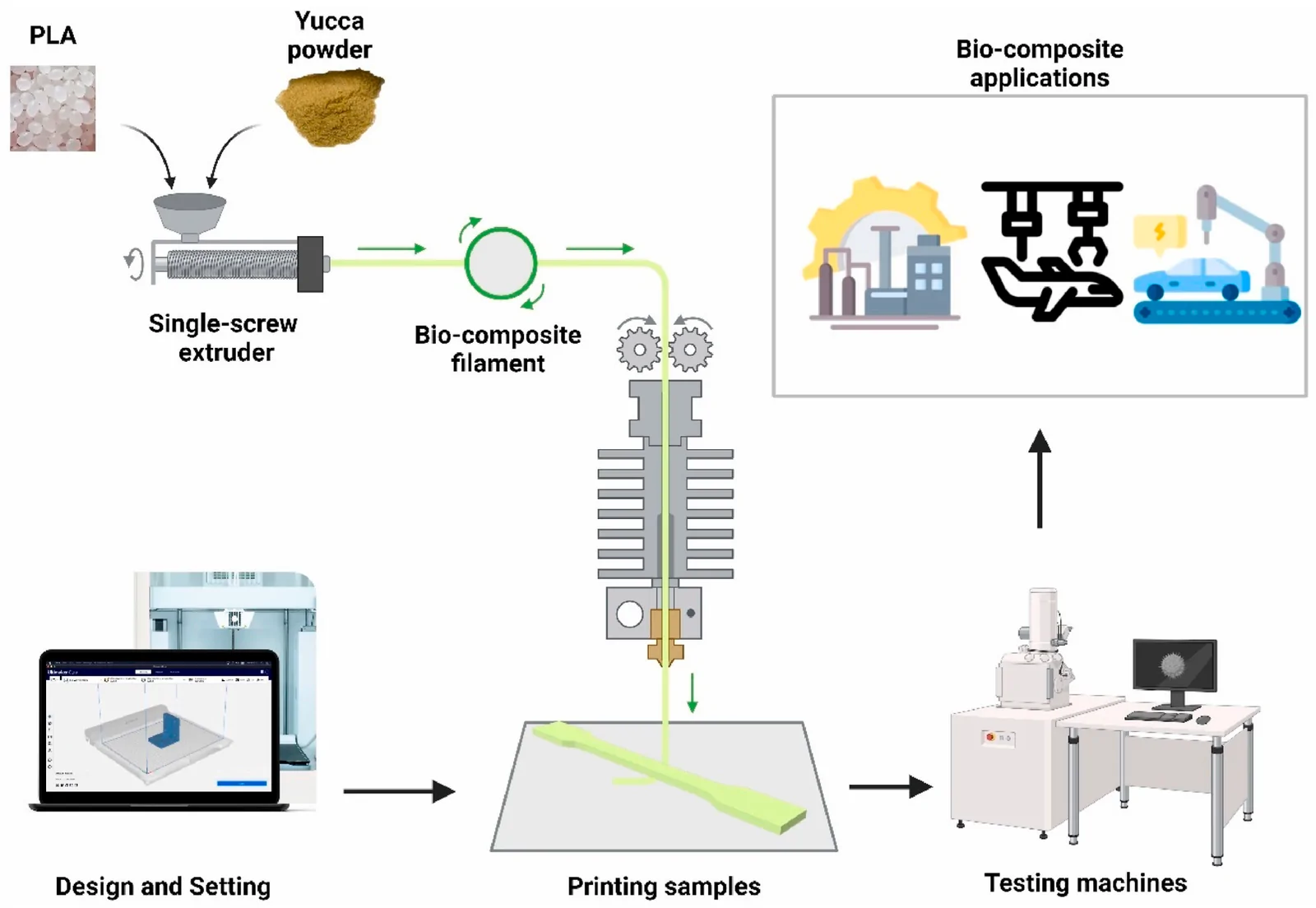
Typical example showing the processing of filament, analysis, and applications of PLA reinforced with yucca fiber. Reproduced from [89]. Creative Commons CC-BY.

FDM printing requires polymer filaments. NFRPC filaments are generally prepared through extrusion processing. PLA and the fibers can be fed simultaneously into a hopper and extruded together to produce a filament with controlled thickness. Alternatively, NFRPCs can be processed separately to produce composite pellets, which are then further processed to develop the filament. As summarized in Table 5, most of the NFRPC filaments are produced in a single-screw extruder. Most FDM printing instruments require filaments with diameters of 1.75 mm. However, the diameter of reinforced composites may vary due to increased viscosity and inconsistent flow during extrusion caused by fiber properties, including concentration and size distribution. Proper optimization is key to attaining consistent fiber diameters. Figure 5 illustrates a typical example for producing PLA/yucca powder filament, where both PLA and the powder are extruded together to produce a filament, which can then be used for printing [89].

Table 5 summarizes various natural fibers used in developing PLA-based filaments for FDM printing. The sizes of the fibers, concentrations, and nozzle sizes are summarized. In most studies, the targeted filament of 1.75 mm was developed. For FDM printing of NFRPCs, the nozzle size is important to allow continuous flow without discontinuities and blockage. Different nozzle sizes were used to print various NFRPCs. Larger nozzle sizes accommodate higher fiber loadings and fibers with larger sizes. The tabulated studies focused on investigating the influences of printing parameters [90,97], fiber extraction methods [89], fiber treatment, and loading [92,93] on material properties of printed composites. Despite the considerable progress in FDM printing of NFRPCs, future research studies should move beyond the optimization of individual variables toward a comprehensive understanding of processing–structure–property relationships in these materials. The correlation between printing-induced porosity and interlayer bonding and the tribological, mechanical, and thermal (e.g., heat distortion temperatures) properties of these materials should also be investigated. Moreover, the long-term durability of FDM–printed NFRPCs under environmental stresses such as moisture, UV, and temperature variations should also be investigated. Long-term durability studies are crucial for translating NFRPCs from laboratory-scale research to industrial applications because their initial material performance does not reflect their ability to maintain their functionality throughout their service life. Furthermore, these studies may become significant in establishing performance benchmarks and developing appropriate testing standards for these materials. This is a critical research gap in many NFRPCs, as this is an imperative step in bridging the gap between laboratory-scale material and industrial deployment.

### 5.2. Mechanical Properties of 3D-Printed NFRPC Filaments

Natural fibers are ideal reinforcing materials suitable for improving the mechanical properties of FDM-printed PLA composites. Generally, FDM-printed polymer matrices tend to exhibit inferior mechanical properties due to voids, weak inter-layer adhesion, thermal stress, and other factors. Reinforcing polymer matrices with natural fibers is a widely used strategy to enhance their mechanical properties. Numerous studies have investigated how natural fibers affect the mechanical properties of FDM-printed PLA. Several factors influence the mechanical properties of printed NFRPCs, which can be divided into two categories: PLA–natural fiber properties and printing parameters. The PLA–natural fiber properties are general factors that affect the mechanical properties of any fiber-reinforced polymer composite. These include fiber loading, interfacial adhesion between PLA and the reinforcing fiber, dispersion and distribution in the continuous PLA matrix, fiber extraction method, mechanical properties of the reinforcing fibers, fiber size and aspect ratio, and fiber loading. The following section provides an overview of how these factors influence the mechanical properties of printed PLA.

(i) PLA–Natural fiber interaction properties

**Figure 6:**
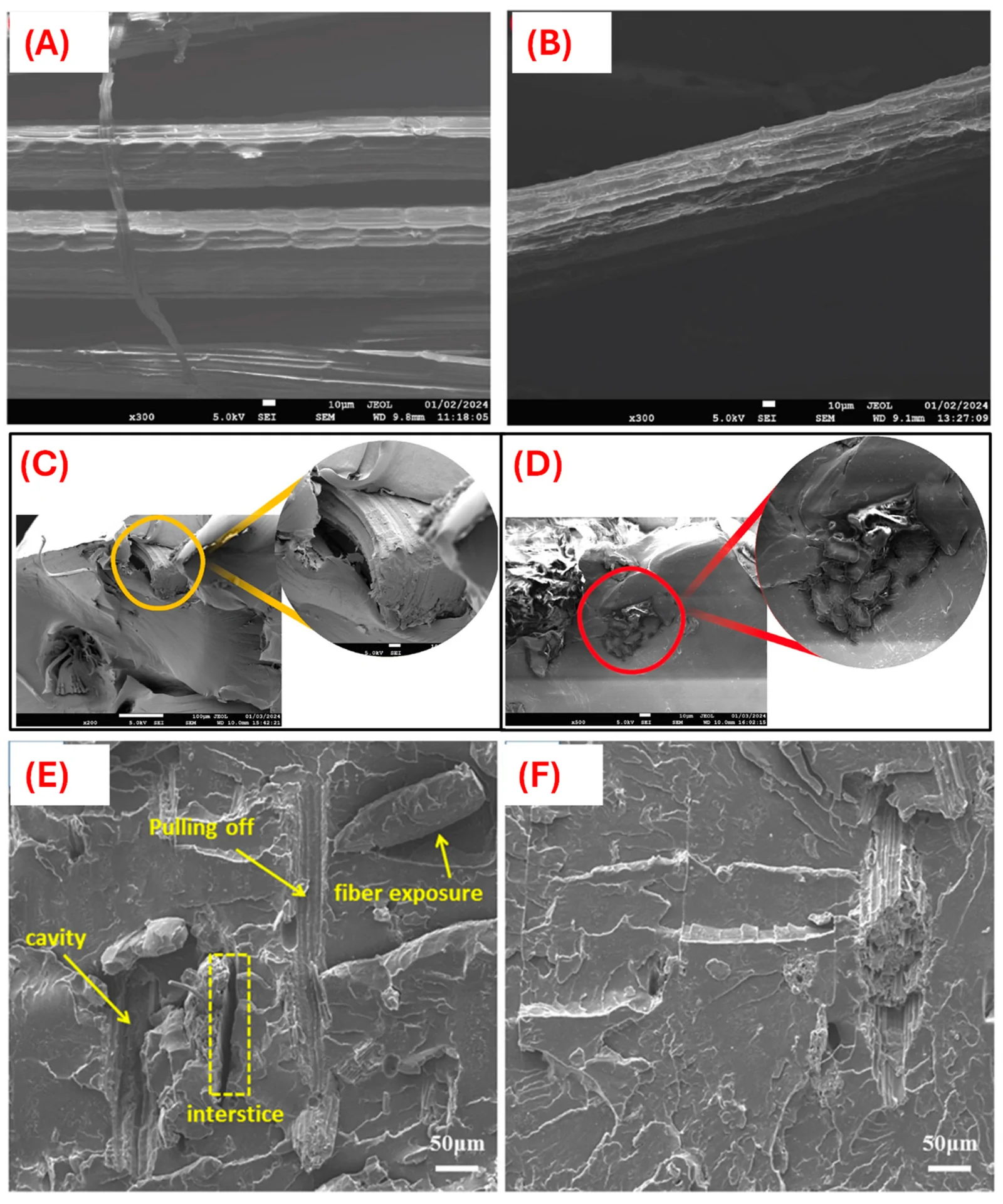
SEM micrographs of (**A**) yucca fiber extracted using water retting and (**B**) yucca fiber extracted using the traditional method. Biocomposites reinforced with 1% yucca fibers (**C**) extracted with water retting and (**D**) the traditional method. Reproduced from [89]. Elsevier, Creative Commons CC-BY. SEM micrographs of (**E**) PLA/5 wt. % LF without PLA-g-MA and (**F**) with 5 wt. % PLA-g-MA. Reproduced with permission from [70], John Wiley and Sons, 2020.

Natural fibers can be extracted through various methods, as summarized in Table 1. These methods usually affect fiber morphology and surface properties, such as roughness, which in turn affect their interaction with the polymer matrix. The extraction methods used to extract *Yucca* fibers from *Yucca treculeana* leaves affected the mechanical properties of printed PLA/Yucca fiber composite filaments [89]. The fibers were extracted by the traditional method and the wet-retting method. The fibers extracted using the traditional method had rougher surfaces (Figure 6A,B). Usually, rough surfaces of the fibers with numerous ridges and micro-grooves provide efficient interfacial adhesion and mechanical interlocking with the polymer matrix [108]. As shown in Figure 6C,D, the scanning electron microscopy (SEM) micrographs of fibers extracted with the traditional method showed excellent adhesion to the PLA matrix, whereas the water-retting extracted fibers showed voids and poor interfacial adhesion with the PLA matrix. As a result, 3D-printed composites containing 1 wt. % of the traditional method-extracted fibers exhibited the tensile strength of 61.06 MPa, Young’s modulus of 1316 MPa, and elongation of 4.64%, which were higher compared to printed composites with 1 wt. % of water-retting extracted fibers that showed tensile strength of 49.73 MPa, Young’s modulus of 1201 MPa, and elongation of 4.14%. These findings indicate that the extraction method significantly affects fiber morphology, which in turn influences interfacial adhesion with the PLA matrix and mechanical properties.

Natural fibers are hydrophilic in nature due to the abundance of –OH groups. Consequently, they show poor or weak interaction with polymer matrices, which can lead to mechanical failure [109]. Chemical treatment of the fibers enhances interfacial adhesion with the PLA matrix, thereby improving stress transfer and the mechanical performance of FDM-printed composites. Usually, natural fibers are chemically treated (e.g., alkali treatment or silanization) to improve adhesion and wetting with the PLA matrix, which subsequently leads to efficient stress transfer from the matrix to the fibers. Reported studies show improved mechanical properties in printed PLA containing chemically treated fibers. In addition to chemical treatment, the fiber loading (with/without chemical treatment) also dictates the mechanical behavior of printed PLA composites. Somsuk et al. [93] investigated the effects of fiber treatment and size, as well as fiber loading, on tensile and flexural properties of 3D-printed PLA/RH and PLA/rice straw (RS) composites. The authors noticed that these parameters influence the mechanical properties of printed NFRPCs. For instance, printed composites containing alkali-treated fibers had higher tensile and flexural strength than untreated composites, although strength decreased when the concentration increased from 5 to 10 wt. %. At higher concentrations, the benefits of surface modification can diminish because fiber clustering and clump formation can act as stress concentrators that eventually lead to mechanical failure. In addition, fiber mesh size played a role in achieving better mechanical performance. Smaller fibers tend to disperse better within the continuous matrix and transfer stress more efficiently; however, they also tend to agglomerate, which usually adversely affects mechanical properties. Despite chemical treatment, optimizing fiber loading remains crucial, as higher loadings adversely affect the mechanical behavior and performance of printed PLA. Silane (KH550)-treated short basalt fibers enhanced the tensile and flexural strength of FDM-printed PLA, with improvements observed as fiber loading increased from 5 to 20 wt. % [110]. Similarly, Jamadi et al. [111] investigated the combined effect of silane and alkali treatment of kenaf fiber on the mechanical properties of 3D-printed PLA. The alkali concentration was fixed at 6%, while the silane concentration varied from 0.5 to 2 wt. %. Among the investigated formulations, the composite with 1% silane showed the highest tensile and flexural strength and moduli, highlighting the importance of optimizing treatment conditions to maximize interfacial bonding and mechanical performance. Comparable findings were reported by Srivastava et al. [92] for PLA/hemp composite filaments containing 2.5 and 5% of chemically treated hemp fibers. The printed composites showed higher Young’s modulus, strain, and tensile strength than neat PLA. For example, the Young’s modulus of PLA was 3.2 GPa, while PLA/hemp (2.5%) and PLA/hemp (5%) were 3.76 and 4.09 GPa, respectively. Likewise, tensile strength improved by 29.6% and 21.7% for the 2.5 wt. % and 5 wt. % fiber loadings, respectively. These findings indicate that surface treatment can significantly enhance fiber reinforcing efficiency in FDM-printed PLA composites. However, careful optimization of both treatment conditions and fiber loading is necessary to balance printability and mechanical performance.

Fiber loading plays a critical role in determining the mechanical properties of printed NFRPCs. Excessive fiber loadings can adversely affect the mechanical response of NFRPCs. For example, Somsuk et al. [93] reported a reduction in mechanical properties when the fiber loading was increased to 10 wt. %. Similar findings were reported, where mechanical properties decreased when 10 wt. % of untreated or alkali-treated hemp, flax, and pineapple leaf fiber (PALF), with respect to neat PLA [102]. Higher concentrations are associated with agglomeration, insufficient matrix–fiber interactions, and increased void formation of the fibers, which could result in inefficient stress transfer and mechanical failure. Therefore, optimum fiber loading is required to maximize the reinforcing effect while minimizing processing-induced defects. Mansingh et al. [105] demonstrated the significance of low fiber loadings on the mechanical response of printed PLA reinforced with PALF. The authors evaluated the effect of PALF loading and alkali treatment on the mechanical properties of 3D-printed PLA. The fiber’s (treated or untreated) concentration was varied from 1, 3, to 5 wt. %, which were relatively low compared with studies employing loadings of up to 10 wt. % [93,102,104]. Both treated and untreated PALF resulted in significant enhancements in tensile strength, modulus, and elongation at break and flexural strength and modulus, as well as fracture toughness in both treated and untreated fibers compared with neat PLA across the investigated concentrations, as summarized in Table 6. These findings suggest that the effectiveness of the fibers in improving the mechanical properties of printed NFRPCs does not necessarily depend on maximizing fiber content; rather, it depends on maintaining adequate fiber dispersion, interfacial adhesion, and wetting. Overall, the findings highlight the need to optimize fiber loading alongside fiber treatment and printing parameters. 

Adding compatibilizers also enhances interfacial compatibility between PLA and the loaded fibers, improving the mechanical properties of the printed composite material. Jing et al. [70] showed that adding 5 wt. % of PLA-g-MA into PLA/lemongrass fiber (LF) can maintain a balance in the mechanical properties of PLA/LF (5 wt. %). The mechanical properties of PLA/LF showed a decreasing trend in tensile strength, impact modulus, and flexural strength compared with neat PLA. For example, the tensile strength decreased from 59.6 MPa (PLA) to 45.1 MPA (PLA/LF), whereas the flexural strength decreased from 98.3 MPa (PLA) to 78.2 MPa (PLA/LF). Upon addition of PLA-g-MA at 5 wt. %, the tensile and flexural strengths reached 54 MPa and 96.2 MPa, respectively. The impact strength was 2.5 KJ/m^2^, comparable to neat PLA (2.6 KJ/m^2^) and PLA/LF (2.1 KJ/m^2^). The flexural modulus also increased by 27% and 50% for PLA/LF and PLA/LF/PLA-g-MA, respectively, with respect to neat PLA. With a compatibilizer such as PLA-g-MA, interfacial compatibility between PLA and LF fiber is high, as shown by the absence of obvious voids, gaps, or holes (Figure 6E,F). However, PLA/LF composites without a compatibilizer show fiber pullouts, cavities, and interstices, confirming poor interfacial compatibility.

(ii) Printing parameters

One advantage of 3D printing is its versatility in fabricating specimens. Printing parameters can be tailored to achieve a printed specimen with enhanced mechanical properties. Several printing parameters, such as infill parameters (e.g., density and pattern), printing speed and temperature, build orientation, layer height, and number of walls, can have an effect on the mechanical properties of the printed part. The influence of printing parameters on the mechanical properties of FDM-printed NFRPCs has been widely investigated [63,87]. For example, Soni et al. [90] investigated the effect of raster angle on PLA/Tamarindus indica seed containing 20 wt. % filler and reported that increasing the raster angle from 0° to 60° enhanced both tensile strength and tensile modulus, followed by a decline at 90°. In contrast, the flexural response exhibited an opposite trend, with both flexural strength and modulus decreasing as the raster angle increased from 0° to 90°. These findings demonstrate that the optimum raster angle may depend on the type of mechanical loading, since the filament orientation that favors tensile load transfer may not necessarily provide the most effective resistance to flexural deformation. In another study by Agaliotis et al. [97], a different trend was observed: the tensile modulus and strength of PLA/henequen (1 wt. %) decreased as the raster angle increased from 0° to 45° and 90°. This contrasting behavior indicates that raster angle cannot be considered independently of composite formulation and processing conditions. Differences in fiber type, morphology, loading, surface treatment, and printing parameters may alter interfacial adhesion, fiber orientation, interlayer bonding, and ultimately the mechanical response. The interaction between raster angle and fiber content further shows the importance of optimizing material and printing parameters simultaneously. Figueroa-Velarde et al. [104] investigated the combined effect of agave fiber loading and raster angles (−45°/45° and 0°/90°) on tensile properties of PLA/agave fiber composites. They observed a decrease in tensile strength with increasing agave fiber loading for both raster configurations, with a pronounced reduction at high fiber loadings (5 and 10 wt. %). Similarly, tensile modulus declined, especially at high fiber content. These observations suggest that increasing natural fiber loading does not necessarily translate into improved mechanical performance in FDM-printed composites. At higher loadings, fiber agglomeration, inadequate wetting, increased void formation, and disruption of interlayer bonding may outweigh the potential reinforcing effect of the fibers. Overall, these studies indicate that the mechanical performance of 3D-printed NFRPCs is governed by the coupled effects of material formulation and printing parameters rather than by any individual parameter. Consequently, to achieve effective stress transfer and interlayer bonding while minimizing defects and anisotropy, optimization of fiber loading, fiber treatment, raster angle, and other processing conditions should be considered simultaneously.

**Figure 7:**
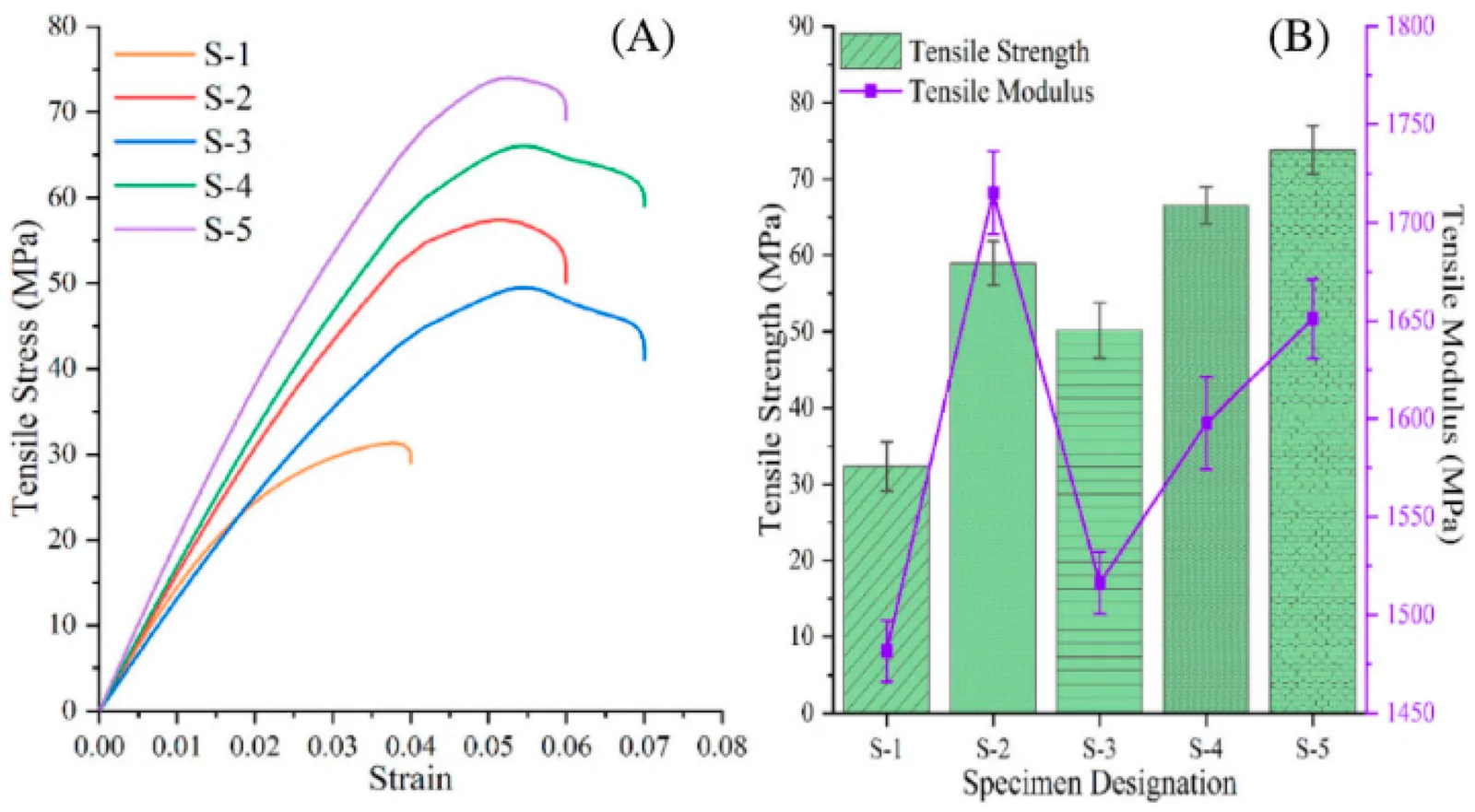
(**A**) Tensile stress–strain curves and (**B**) tensile strength and modulus of printed specimen. Reproduced with permission from [94]. John Wiley and Sons, 2024.

Although different printing parameters influence the resulting mechanical properties, the composite specimen’s build structure can also affect them. Vinod et al. [94] investigated the effect of build structure on the mechanical properties by employing a novel strategy to improve the mechanical properties of 3D-printed NFRPCs. The method used layer-by-layer (0.75 mm per layer) stacking of PLA/nona and PLA/soy composites to develop the 3D-printed specimen. They developed different configurations from PLA filaments with a fixed concentration (6% vol) of either nona or soy fiber. One composite system comprised four layers of PLA/nona (S1), and another comprised four layers of PLA/soy (S2). The other three composite specimens comprised alternating layers of (PLA/nona–PLA/soy–PLA/nona–PLA/soy) (S3), (PLA/nona–PLA/soy–PLA/soy–PLA/nona) (S4), and (PLA/soy–PLA/nona–PLA/nona–PLA/soy) (S5). Figure 7 depicts a typical stress–strain and tensile modulus plot of the developed layered composites. As shown, the S5 composite reached the highest tensile strength and modulus, which were fairly high compared to most reported studies [90,97,104]. Additionally, the S5 composite reached the highest modulus of toughness (3178.3 KJ/m^3^) and modulus of resilience (395.54 KJ/m^3^). Xing et al. [106] developed FDM-printed PLA/bamboo yarns, PLA/flax-C, and PLA/flax through in situ impregnation of the polymer matrix into natural yarns, where both PLA filament and the yarns were simultaneously fed into the nozzle and printed. The printed composites showed higher strength than neat PLA, with PLA/flax yarns showing the highest tensile and flexural strength, as well as the highest flexural modulus. Table 7 summarizes the mechanical properties of other reported FDM-printed NFRPCs [91,110,112,113,114,115].

Mechanical properties of FDM-printed PLA/natural fiber are governed by filler loading, matrix–fiber interactions, and printing parameters. Among these, improved interfacial adhesion between PLA and natural fiber is important for maximizing mechanical improvements because it promotes efficient stress transfer between the PLA and natural fibers. However, these benefits must be considered along with fiber loadings and printing conditions. For example, Soni et al. [90], the combined effect of fiber treatment and increasing raster angle up to 60° provides a synergy for enhanced mechanical properties. Interestingly, a layered fabrication of printed composite seems to provide significantly high mechanical properties, albeit this was shown to depend on layer configuration [94]. These observations demonstrate the benefits of architectural design as an additional factor to maximize the mechanical performance of printed NFRPCs. Collectively, the summarized findings indicated that improved mechanical properties of NFRPCs can be attained by enhancing the PLA–natural fiber interfacial interactions, appropriate fiber loadings and dispersion, and optimization of fiber morphology and printing parameters. These are key parameters to be considered when developing 3D-printed NFRPCs, and they should be considered independently. In combination with optimized printing parameters, the synergistic effect of composite filament comprising excellent matrix–fiber interactions and good fiber dispersion can be obtained, which would subsequently result in enhanced mechanical properties. From the summarized results, several considerations emerge.

(i) The concentrations of natural fibers for better printability and enhanced mechanical properties can be obtained at relatively low-to-moderate fiber loadings, particularly below approximately 5 wt. %. This range often provides favorable performance due to better dispersion, fewer agglomerates, and fewer voids. However, this should not be considered as a universal optimum, since appropriately treated fibers and optimized printing parameters can allow higher loadings while maintaining a balance between printability and mechanical performance.
(ii) The extraction methods affect the surface properties of the natural fibers and increase their roughness, which subsequently improves adhesion with the PLA matrix. Furthermore, chemical treatment of natural fibers, or incorporation of compatibilizers such as PLA-g-MA, can significantly improve the interfacial properties. These strategies have been reported to improve matrix–fiber adhesion and consequently mechanical performance. However, chemical treatment can alter the structural integrity and strength of natural fibers, which may reduce their reinforcing efficacy in PLA.
(iii) Optimization of printing parameters, such as raster angle, layer configuration, and other printing conditions, is crucial for achieving improved mechanical performance of FDM-printed NFRPCs. However, optimum printing conditions may vary with fiber loading, surface treatment, and fiber orientation. Thus, the printing parameters and material formulation should be optimized simultaneously rather than independently.

## 6. Tribological Properties

Tribological properties are essential because they determine wear resistance, self-lubrication ability, and coefficients of friction. In addition, they are important in predicting the service life of the material [109]. These properties indicate the load-bearing capacity, damping capacity, and application suitability of materials in harsh environments subjected to continuous friction. Wearing behavior can be evaluated based on wear mechanisms including corrosive wear, abrasive wear, and adhesive wear. The printing parameters have been shown to influence tribological properties. Printing-induced voids and interlayer interfaces can strongly influence the tribological properties of FDM-printed samples, as these microstructural features may affect surface roughness and wear rates. Increasing the infill density reduces the coefficient of friction. At higher infill densities, porosity decreases and smoothness increases, contributing to an overall decrease in the coefficient of friction. Sanches et al. [112] noticed this behavior in FDM-printed PLA containing bamboo fibers (10%), where increasing the infill density to 100% reduced the coefficient of friction to 0.63. Other printing properties such as layer thickness and print orientation were shown to affect the coefficient of friction. Layer thickness has been shown to affect the coefficient of friction [109,112]. Reducing layer thickness may significantly improve interlayer adhesion and minimize microstructural voids between the layers and improve tribological properties [116]. The tribological properties of FDM-printed hemp-containing PLA composites showed increased frictional force and height loss with increasing load from 10 to 30 N. The observed increase in frictional force and height loss may indicate wear of the printed composites [114].

Pre-treatment of natural fibers under alkali conditions improves wear resistance and reduces the coefficient of friction of the biocomposites. Therefore, treatment of natural fibers may not only enhance interaction with the PLA matrix but also improve the tribological properties, such as decreasing the wear rate [117]. Improving tribological properties, such as wear resistance of FDM-printed NFRPCs, is important to expand their application, particularly for applications such as automotive components and orthopedic implants, where the materials are often subjected to friction. For such applications, incorporating alkali-treated natural fibers into PLA, combined with higher infill densities and reduced layer thicknesses, may provide a synergistic effect. However, future research should focus more on investigating processing–structure–property relationships governing improved tribological properties, such as reduced wear rates.

## 7. Life Cycle Assessment of FDM-Printed NFRPCs

Additive manufacturing, particularly FDM, has emerged as a key manufacturing technique for processing thermoplastics and their composites. FDM-printed NFRPCs are promising sustainable materials for advanced, high-performance applications. However, to fully integrate these materials along with the 3D printing process, LCA studies are imperative to understand their environmental sustainability. Currently, few studies integrate LCA into FDM printing of NFRPCs, highlighting the need for further research in this area. Table 8 summarizes the LCA studies on FDM-printed NFRPCs.

Cozzolino et al. [118] evaluated the environmental impact of printing 1 kg of PLA containing 2 and 10% of basalt powders and short hemp fibers. Across all evaluated impact categories, environmental impact decreased with the incorporation of 10% basalt and hemp fibers. For instance, the total contribution of PLA to GWP reduced from 14 kg CO_2_-eq (PLA) to 12.8 and 12 kg CO_2_-eq by incorporating 10% of basalt and hemp fibers, respectively. PLA production contributes more to CO_2_ emissions than the 3D printing process. However, introducing basalt and hemp fibers reduces GWP, significantly lowering the total contribution to GWP. Incorporating the two fillers at 10% slightly reduces the total contribution to AP and EP. Considering energy demand, the 3D printing process consumes a high amount of energy, showing the highest cumulative energy demand (CED) of 35 MJ to obtain 1 kg of printed material. 3D printers comprise a printer table, motors, and nozzle heating, which drive high energy consumption. In contrast, Enarevba et al. [119] evaluated the energy and carbon-related impacts of 3D printing filaments of PLA containing hemp materials (straw, hurd, and fiber). Although the composite filament generally exhibited lower GWP contributions than neat PLA, the environmental performance varied considerably depending on the type of hemp material used. The CED single-score results indicated that PLA/hemp fiber filaments required more energy (73.20 MJ) than neat PLA (66.08 MJ), PLA/hemp straw (29.75 MJ), and PLA/hemp hurd (36.13 MJ). Additionally, the CED impact of PLA/hemp fiber increased with increasing the loading concentration of hemp fibers, while for other composites, the CED impact decreased with loading concentration from 5 to 15%. PLA also contributed more to GWP with 3.1 kg CO_2_-eq than the composites; however, PLA/hemp fiber filament contributed more to GWP with 2.97 kg CO_2_-eq than PLA/hemp straw (1.37 kg CO_2_-eq) and PLA/hurd (1.47 kg CO_2_-eq). Moreover, PLA/hemp fiber filaments had higher impacts than PLA in categories including freshwater and marine eutrophication, water consumption for human health, terrestrial and aquatic ecosystems, and ozone depletion. These findings indicate that the environmental impacts of NFRPCs do not automatically reduce, and they may differ from one system to another. Overall, the summarized studies demonstrated that the environmental sustainability of FDM-printed NFRPCs is influenced by both material composition, fiber/filler type, and loading concentrations. Replacing part of the PLA matrix with natural filler can reduce certain environmental impacts; however, this benefit may be offset by additional energy requirements and processing steps. Thus, the use of renewable energy, particularly for 3D printing as it consumes significantly high amounts of energy, may contribute to reducing the environmental footprint of these materials. In addition, more LCA studies on 3D printing of NFRPCs should be conducted to systematically evaluate the performance of different natural fibers, fiber extraction, and treatment methods, filament production, printing, and end-of-life scenarios. Such studies would be essential in providing a more comprehensive understanding of the environmental trade-offs related to FDM-printing of NFRPCs and help guide the development of more sustainable manufacturing routes.

## 8. Discussion

**Figure 8:**
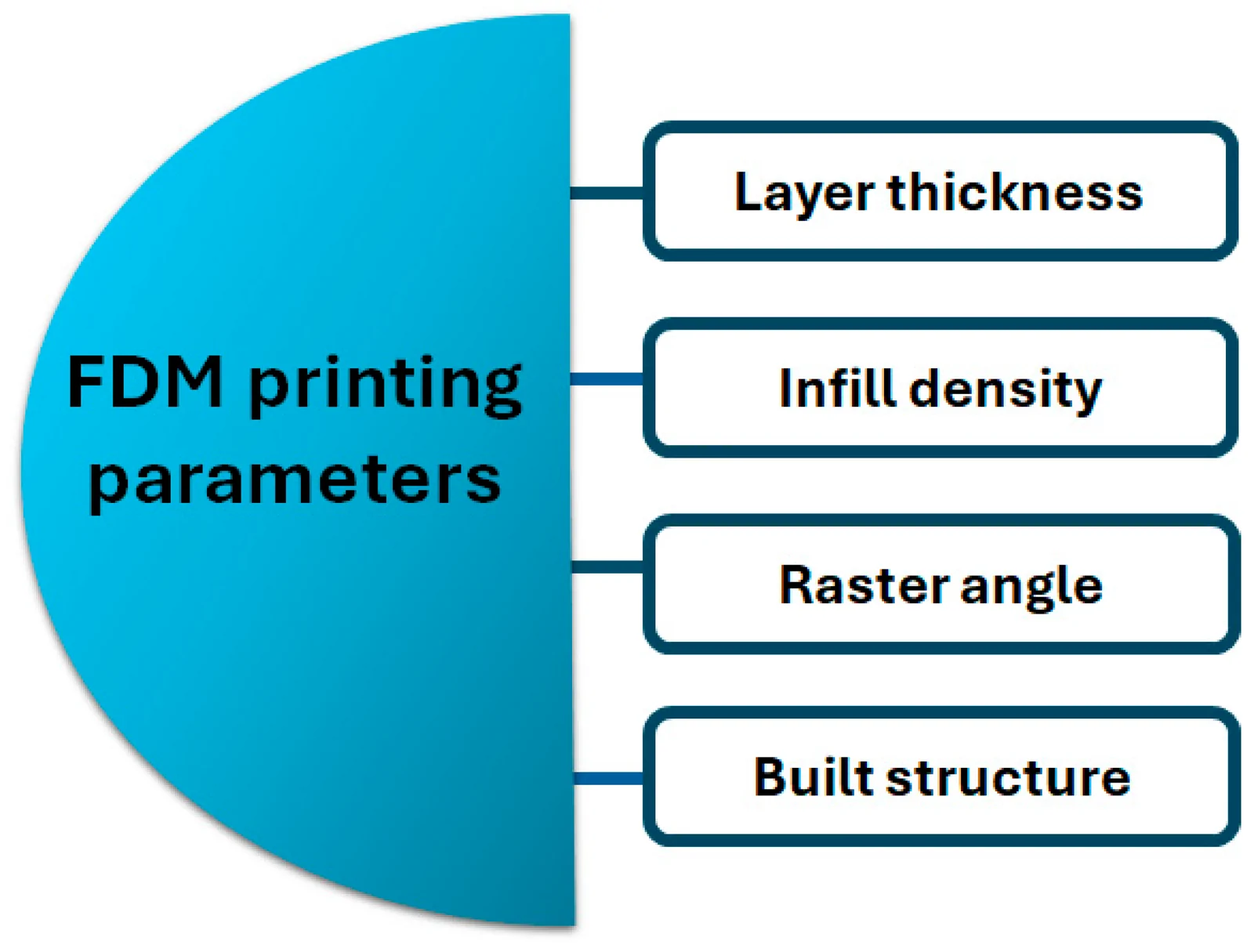
FDM printing parameters influencing the mechanical and tribological performance of NFRPCs.

NFRCs have gained enormous attraction in various application fields such as automobiles, aeronautics, packaging, construction, and so forth. Furthermore, the AM of these composites has gained substantial momentum in designing unique, intricate, and advanced materials that are unattainable with traditional polymer processing techniques. In this review, a brief overview of advancements in developments and applications of FDM-printed NFRPCs was provided. These materials hold huge potential for utilization across various application fields, primarily because of various key merits they exhibit, which include complete biodegradability/compostability, environmental friendliness, and non-toxicity. Additionally, a significant research interest in FDM printing of NFRPCs emanates from the good printability of PLA compared to many other commonly known polymers, and also the abundance of natural fibers at low to zero costs, as most of them are regarded as low-value and underutilized materials, especially those classified as agricultural waste. However, due to their hydrophilicity, there is limited interfacial adhesion with polymers like PLA, resulting in morphologies comprising voids, gaps, and agglomerated fibers in a continuous matrix, which results in mechanical failure. Because this type of composite is intriguing for various applications, its mechanical properties must meet each application’s specifications, as most applications depend primarily on the composites’ load-bearing capacity. Consequently, morphological properties should lead to satisfactory mechanical performance. This review shows that better mechanical properties in 3D-printed NFRPCs result primarily from enhanced interfacial adhesion between the reinforcing fiber and the PLA matrix. Thus, modifying the fibers is crucial for improved interfacial interactions with PLA. Alkali treatment is widely used, possibly because it increases surface roughness, which improves mechanical interlocking and wettability by the PLA matrix. Although chemical treatment improves compatibility between natural fibers and the PLA matrix, it may also degrade the fibers, alter structural integrity, and reduce strength. Other modifying agents, such as silane, can be used to modify natural fibers. However, LCA studies have shown that these treatments contribute adversely to the environment, highlighting the need to seek “greener” alternative methods for modifying the fibers. This is an important research gap that should be addressed, particularly when considering the FDM printing of NFRPCs.

(iv) The inclusion of compatibilizers is also promising for achieving desired morphologies and mechanical properties, as they act as anchoring agents between PLA and hydrophilic fibers and provide efficient stress transfer under mechanical load. Fiber loading also proved important for enhanced mechanical response, especially at low concentrations, where improvements were greatest compared to higher loadings. Generally, at low concentrations, there is better dispersion and/or distribution of the fibers with no clumps or agglomerates. Ideally, larger fiber amounts in the composites are desired because of the potential to reduce the material costs. However, for FDM printing, nozzle size limits higher fiber loadings in the filaments. Printing parameters are also key in improving the mechanical performance of printed NFRPCs. Printing conditions, such as raster angles, have been shown to influence mechanical properties. However, further investigation into the effects of other printing conditions is still required to identify a broad range of parameters that can effectively enhance the material properties. Few studies have examined the tribological properties of FDM-printed NFRPCs; therefore, future research should also focus on investigating these properties. Furthermore, improving the tribological properties of FDM-printed NFRPCs depends on the targeted application and may not always be necessary. Figure 8 summarizes the FDM printing parameters affecting the mechanical and tribological properties of FDM-printed NFRPCs. Optimization of these parameters is key to achieving FDM-printed NFRPCs with improved mechanical and tribological properties. Other printing parameters, such as infill patterns, shell thickness, printing temperatures and speeds, among others, should also be investigated.

## 9. Limitations and Recommendations

FDM printing of NFRPCs is promising for developing sustainable, green composites with complex and customized geometries. Nevertheless, some challenges can limit the use of this technology in developing environmentally benign NFRPCs. Generally, PLA is a brittle polymer with extremely poor toughness and impact resistance. Thus, printed PLA parts may be susceptible to fragility. PLA with added toughening agents may be suitable to produce printed NFRPC items with enhanced toughness. Inconsistent fiber size and distribution may cause inconsistent filament diameter due to uneven flow and changes in viscosity during extrusion. This can also affect the printing process and print quality, as inconsistencies in layer thickness can reduce quality. Optimized size distribution may help achieve constant flow and viscosity and consistent filament diameters. Another factor to consider is filler loadings, as high concentrations of natural fibers may cause nozzle blockage and discontinuities during printing. Therefore, larger nozzle diameters of ~0.8–1 mm could be effective. In addition, higher fiber content may affect the appearance of the printed part, especially when aesthetics are important. Ironing during printing may improve surface finish and appearance. Because the mechanical properties of printed NFRPCs depend on interfacial adhesion between the fibers and the PLA matrix, using chemicals to treat the fibers may raise concerns, especially at the industry level, given the solvents involved in modification. In addition, industrial-scale modifications may be costly because they require specialized reactors and consume significant energy. This necessitates green methods for modifying the fibers that are energy-efficient and inexpensive. Compatibilizers, such as PLA-g-MA, can be a viable option to improve compatibility in NFRPC systems without the need to modify the fibers. However, grafting PLA with MA may be toxic to human health and the environment. In this case, LCA studies may help identify the environmental impacts and energy consumption of this process. Future studies should also focus more on investigating printed PLA containing hybrid fillers (i.e., natural fiber + other fillers). A combination of two natural fibers, or natural fibers with another filler, provides unique properties that cannot be obtained with respective individual fillers. FDM-printed hybrid NFRPCs can be developed with enhanced properties such as flame retardancy, mechanical properties, thermal stability, and conductivity.

Another challenge with NFRCs is their inherent flammability. NFRPCs are also flammable, making even their FDM-printed parts flammable. This indicates the need to investigate hybrid composites containing natural fiber and flame-retardant materials [2]. Understanding the interactions and properties of such hybrid systems will clarify which flame-retardant materials and concentrations are needed to balance printability, mechanical performance, and flame retardancy. This will further broaden the use of NFRPCs in advanced applications. Hygroscopic aging in NFRPCs requires extensive intervention, as these materials tend to absorb excessive moisture that weakens the interfacial adhesion and subsequently deteriorates mechanical properties. Generally, NFRCs perform poorly when exposed to environments with varying humidity and temperatures. Natural fibers are hydrophilic in nature. Similarly, PLA has a moisture absorption capacity, presenting a challenge when combined with natural fibers [2,8,120]. Therefore, it is crucial to investigate the influences of moisture absorption at varying relative humidity on printed NFRPC interfaces, interlayer bonding in printed parts, and overall load-bearing properties of 3D-printed NFRPCs. The prototypes should also be developed from these biocomposites, as this would clarify the application path for these materials. Because numerous properties and parameters require optimization to maximize mechanical performance, artificial intelligence (AI) tools and machine learning techniques may help reduce wasted resources and time for physical optimization. AI and machine learning offer considerable potential to advance FDM-printed NFRPCs through data-driven optimization of material formulation, printing parameters, mechanical performance, LCA, and application-specific design. Moreover, research studies rarely report the cost performance of these materials; therefore, future research should systematically investigate cost performance alongside mechanical and other material performance to enable meaningful cost performance comparisons.

## 10. Applications

**Figure 9:**
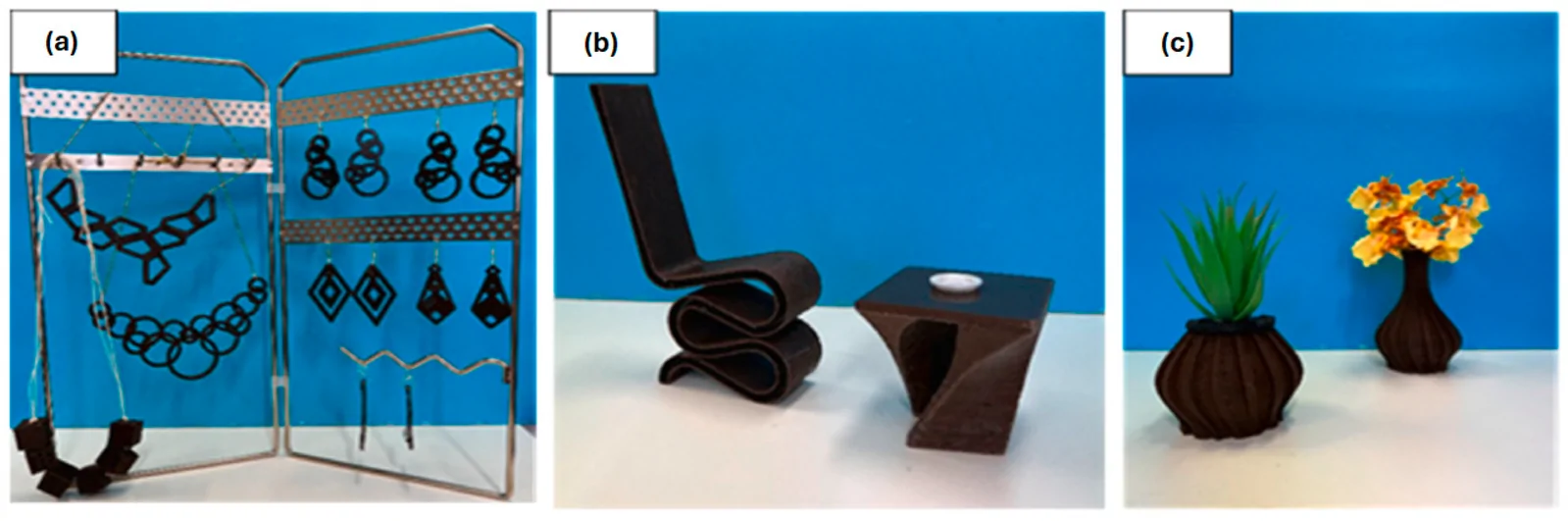
FDM-printed prototypes made of PLA/HSP composites: (**a**) jewellery, (**b**) home furniture, and (**c**) ornamental. Reproduced from [121]. Elsevier. Creative Commons, CC-BY.

Generally, FDM provides flexibility for fabricating customized and intricate components from NFRPCs. The potential utilization of a material strongly depends on key requirements for a particular application. For example, mechanical, thermal, tribological, and dimensional stability are among key requirements for most applications. In FDM–printed NFRPCs, understanding these properties is important in projecting their potential applications. FDM-printed NFRPCs can be suitable for various applications, including decorative items and furniture. For example, Alitotta et al. [121] fabricated various prototypes from PLA/hazelnut shell powder (HSP) using FDM. The printed items exhibited a distinctive wood-like aesthetic (Figure 9). The developed materials indicate that these materials can be suitable for developing consumer and household products, such as coasters, towel clips, desk organizers, and holders.

NFRPCs are promising future materials in the automotive sector. Consequently, FDM-printed NFRPCs can also extend to this sector and other engineering applications where lightweight and customized parts, such as panels, holders, and interior accessories, may be considered. However, the suitability of these applications depends on mechanical properties and durability under service conditions. The FDM-printed NFRPCs may be suitable for biomedical applications, such as customized scaffolds. However, their utilization in direct-contact biomedical applications requires careful evaluation, such as cytocompatibility, biodegradability, and regulatory requirements. Therefore, future potential opportunities may be in the development of testing kit cases and other related objects.

The functionality and application scope of FDM-printed NFRPCs can be further expanded through hybridization with functional fillers. For example, the incorporation of carbon nanotubes into PLA/bamboo fiber composites has been shown to impart electrical conductivity and EMI shielding. Such a multifunctional system could potentially broaden the utilization of FDM-printed NFRPCs toward applications requiring these functional properties. Flammability remains a critical limiting factor for wider application of NFRPCs in general. As a result, FDM-printed NFRPCs with improved flame retardancy are important, particularly in automotive, electrical, and other applications requiring stringent fire safety performance. Due to their high flammability, natural fibers can influence the combustion behavior of PLA. Therefore, it is imperative to improve the flame-retardant properties of these composites through appropriate strategies such as hybrid reinforcement with flame-retardant fillers. This could further expand the application potential of FDM-printed NFRPCs. Overall, future research should investigate hybrid NFRPCs incorporating conductive, flame-retardant, or other functional fillers while maintaining printability and load-bearing performance. Lastly, future research should move beyond demonstrating the printability of NFRPCs and prototyping and move toward application-specific validation under realistic service conditions.

## 11. Conclusions

The development of FDM-printed composite materials, particularly from renewable resources, has received considerable attention in developing sustainable future material solutions. This review examined recent developments in the fabrication of NFRPCs using FDM printing, with particular emphasis on mechanical and tribological properties, processing characteristics, and environmental considerations. The reviewed studies demonstrated that NFRPCs can be successfully printed. The mechanical and tribological properties of the printed NFRPCs were shown to be strongly governed by the interplay between fiber loading, matrix–fiber interfacial adhesion, fiber characteristics, and printing parameters. The available LCA studies demonstrated that environmental performance depends on factors such as fiber extraction and treatment, material processing, and energy consumption. Therefore, the use of renewable resources does not necessarily guarantee lower environmental impacts. Overall, FDM-printed NFRPCs demonstrated considerable potential for producing customized, renewable, and sustainable biocomposites suitable for diverse applications.

## Figures and Tables

**Table 1:** Chemical compositions and physical properties of some selected plant fibers [1,2,45,46].

Fiber	Cellulose (wt. %)	Hemicellulose (wt. %)	Lignin (wt. %)	Pectin (wt. %)	Ashes (wt. %)	Wax (wt. %)
Abaca	56–63	15–17	7–9	1	3	3
Agave	68–80	15	5–17	–	–	0.26
Bagasse	28.3–55	20–36.3	21.2–24	–	–	0.9
Corn	47	43.96	4.13	–	2.93	–
Bamboo	26–43	30	1–31	–	–	11
Coconut/coir	32–43.8	0.15–20	40–45	3–4	–	–
Cotton	36–91	3	–	0.6	–	8–9
Oil palm	60–65	–	11–29	–	–	–
Hemp	70–74	0.9	3.7–5.7	0.8	0.8	1.2–6.2
Jute	61–72	18–22	12–13	0.2	0.5–2	0.5
Flax	60–81	14–20.6	0.9–2.3	1.8–2.3	–	1.5–1.7
Kenaf	45–57	8–13	22	0.6	2–5	0.8
Nettle	86	5.4	4	0.6	–	3.1
Sisal	60–78	10–24	8–14	10	–	10–22
Banana	63–67.6	6–19	5–10	3–5	–	11
Pineapple leaf	70–82	–	5–12	–	1	–
Rice husk	35–45	12–25	20	–	–	–
Ramie	69–91	5–15	0.4–0.7	1.9	–	0.3
Softwood	40–45	25–30	26–34	–	–	–
Hardwood	31–64	25–42	14–34	–	–	–

**Table 2:** Summary of natural fiber extraction methods [1].

Method	Description	Advantages	Disadvantages
Chemical Chemical Retting	Chemicals (alkalis or acids) dissolve pectin and lignin.	Quick process; highly efficient; highly scalable; good fiber quality.	Possible fiber damage; high environmental risks.
Biological Dew Retting	Microbial decomposition of fibers while in the field.	Does not use water or chemicals; low cost; low environmental impact.	Lengthy processing time, variable fiber quality, weather-dependent, poor efficiency, and scalability.
Biological Water Retting	Microbial breakdown of pectin in stalks submerged in water.	Low cost; conventional method; moderate efficiency; medium scalability; good fiber quality.	Time-consuming; water-intensive; moderate to high environmental impact.
Biological Enzymatic Retting	Fiber breakdown facilitated by specific enzymes (e.g., pectinases).	Controlled process; low carbon footprint; good fiber quality.	Costly; requires specific operating conditions.
Hybrid Steam Explosion	High-pressure steam treatment followed by rapid decompression.	Quick, high efficiency, preserved fiber strength, moderate scalability.	High cost: pressurized systems required; possible fiber damage; energy demand.
Hybrid Ultrasound-Assisted Retting	Ultrasonic waves accelerate cell wall disruption in a liquid medium.	High efficiency; faster retting; maintained fiber quality; low environmental impact.	High cost; expensive equipment; limited scalability.
Hybrid Microwave-Assisted Retting	Pectin breakdown using microwave heating.	Quick, highly efficient, energy efficient, good fiber preservation, and low environmental impact.	Expensive equipment; limited scale-up.
Hybrid Stand Retting	Pre-harvest field retting with modifications such as chemical or thermal treatments.	Reduced weather dependency; improved fiber quality.	Chemical or energy use may increase environmental impact and cost.

**Table 3:** Effect of extraction methods on morphology and mechanical properties of selected plant fibers.

Fiber	Extraction Methods	Chemical Composition			Mechanical Property	Ref.
		Cellulose (%)	Hemicellulose (%)	Lignin (%)		
Nettle	Chemical retting	81.3.	5.9	2.2	59.9 cN tex^−1^ *	[48]
	Water retting	78.4	9.4	2.8	51.4 cN tex^−1^	
	Microbiological retting with ROO40B	79.6	8.9	3.6	44.8 cN tex^−1^	
	Enzymatic retting with Viscozyme	81.6	12.1	2.1	48.6 cN tex^−1^	
Yucca	Water retting	80.25	13.75	10.45	467 MPa **	[47]
	Traditional	78.06	12.70	10.95	44.74 MPa	
	Chemical	73.95	13.55	7.20	456.23 MPa	
*Schoenoplectus acutus*	Water retting	62.8	20.3	9	302.6 MPa **	[49]
	Chemical	60.2	17.8	6.8	183.4 MPa	
	Manual method	58	25.4	13	254.2 MPa	
Palm grass leaf	Water retting	63.72	2.77	21.24	153.82 MPa ***	[50]
	Chemical	71.93	5.64	20.30	219.11 MPa	
Sisal	Water retting	64	14.5	10.2	514.4 MPa **	[51]
	Chemical	65.2	12.4	11.4	584.91 MPa	

* tenacity, ** tensile strength, *** bundle strength.

**Table 4:** PLA/natural fiber composites patents for use in electronic, construction, and automotive applications.

Patent No. (Year Application Was Granted)	Current Assignee	Natural Fiber	Innovation	Target Application	Ref.
EP3183288B1 (2021)—Polylactic acid composites with natural fibers	Ineos Styrolution Group	Cotton fiber, kenaf fibers, jute fibers, flax fibers, hemp fibers, cellulosic fibers, cotton fibers, sisal fibers, chitin fibers, keratin fibers, and coir fibers.	Introduces a solvent-free process of preparing PLA/natural fiber composites. This method also reduces PLA degradation during processing by limiting residence time at high temperatures	Automotive interior components	[82]
WO2016026920A1 (2015)—Polylactic acid composites with natural fibers	Ineos Styrolution Group	Kenaf fibers, jute fibers, flax fibers, hemp fibers, cellulosic fibers, cotton fibers, sisal fibers, chitin fibers, keratin fibers, and coir fibers.	Introduces a solvent-free process of preparing PLA/natural fiber composites, with improved dispersion and thermal stability	Automotive, construction, electronic	[83]
US8835537B2 (2014)—Natural fiber-based composite material	VTT Technical Research Centre of Finland Ltd.	Peat.	Dried peat is used as a reinforcing agent in biodegradable polymers, including PLA, to reduce moisture absorption	Automotive interior components, packaging	[84]
CN102757628A (2014)—Flame-retardant natural fiber-reinforced polylactic acid composite material and preparation method thereof	Tongji University Mitac Precision Technology Kunshan Ltd.	Amie fiber, jute fiber, flax fiber, hemp fiber, kenaf fiber, sisal fiber, or bamboo fiber.	PLA was modified with nano-additives, antioxidants, and anti-dripping agents; natural fibers were modified with DOPO derivatives. The two were then blended together to produce a flame-retardant bio-composite	Packaging of electronic appliances	[85]

**Table 5:** Summarized plant fibers used to prepare PLA composite filaments, filament diameters, nozzle sizes, and general investigations.

Natural Fiber	Filament Preparation	Filament Diameter (mm)	Nozzle Size (mm)	Fiber Size	Maximum Fiber Concentration	Investigation	Ref.
Tamarindus indica seed	Single-screw extruder	1.75	0.4	100–150 μm	20 wt. %	The effect of raster angles on the mechanical properties of biocomposites was investigated.	[90]
Flax and hemp	Single-screw extruder	2.85	0.6	1 mm	15 wt. %	The effects of fiber loading on mechanical properties and durability of printed composites were investigated.	[91]
Yucca fibers	Single-screw extruder	2.85	0.8	<150 μm	3 wt. %	Fiber extraction methods influenced the properties.	[89]
Hemp fiber	Single-screw extruder	1.75	0.4	<75 μm	5 wt. %	The influence of filler loading on the mechanical properties was investigated.	[92]
Rice husk and Rice straw	Single-screw extruder	1.75	–	100 and 200 mesh	20 wt. %	Fiber treatment with NaOH and the size of the fibers affected the properties of printed composites.	[93]
Nona and soy fibers	Single-screw extruder	1.75	0.6	<50–100 μm	6 vol. %	The stacking sequence of the printed composites influenced the mechanical properties of the 3D-printed composites.	[94]
Tomato stem	–	1.68–1.82	0.8	<150 μm	10 wt. %	The effect of filler loading on the material properties of printed composites.	[95]
Rice husk	Single-screw extruder	1.75	–	<0.5 mm	20%	Effect of alkali treatment on material properties of printed specimens was investigated.	[96]
Henequen	Single-screw extruder	2.78	0.8	90–250 μm	5 wt. %	Printing parameters and fiber concentration influenced the mechanical properties.	[97]
Date palm	Single-screw extruder	1.75	0.4	150–180 μm	5 wt. %	The 3D-printed composites were prepared with a fixed fiber concentration of 5 wt. % fibers.	[98]
Kenaf	Filament extruder	1.75	–	<250 μm	7 wt. %	Effect of fiber loading on fatigue and impact properties was investigated.	[99]
Wood, bamboo, and cork	–	1.75	0.4	–	30 wt. %	The effect of fiber type and infill percentage on the mechanical properties was investigated.	[100]
Kahili ginger fiber	Single-screw	1.75	0.8	90–355 μm	10 wt. %	The effect of alkaline and silane fiber treatment on material properties was investigated.	[101]
Flax, PALF, and hemp	Extruder	1.75	1	~15 and 20 μm	10 wt. %	The effects of the alkali treatment of the fibers on material properties of printed PLA were investigated.	[102]
Hemp	Twin-screw extruder	1.75	1	0.5–1 cm	20 wt. %	The effect of fiber treatment on the properties of 3D-printed were investigated.	[103]
Agave	Twin-screw extruder	1.7	–	–	10 wt. %	Effects of fiber loading and printing parameters were investigated.	[104]
Pineapple leaf	Filament extruder	1.8	1.5	–	5 wt. %	The effects of the alkali treatment of the fibers on material properties of printed PLA were investigated.	[105]
Flax and bamboo	–	1.75	1	–	50.79 and 5.52%	The influences of flax and bamboo yarn on material properties of printed composites were investigated.	[106]
Wood fiber	–	1.75	0.4	–	40 wt. %	The effect of extrusion temperature on mechanical and physical properties was investigated.	[107]

**Table 6:** Mechanical properties and densities of 3D-printed (A) PLA, (B–D: 1–5 wt. %) PLA/PLAF composites and (E–G: 1–5 wt. %) alkali-treated PALF/PLA composites. Reproduced with permission from [105], John Wiley and Sons, 2022.

Sample Name	Tensile Strength (MPa)	Tensile Modulus (MPa)	Flexural Strength (MPa)	Flexural Modulus (MPa)	Elongation at Break (%)	Fracture Toughness (MJ/m^3^)	Density (g/cm^3^)
A	29.5	879.4	32.2	1027.4	4.34	0.6	1.205
B	37.1	1079.1	43.0	1406.0	5.99	1.1	1.218
C	42.3	1311.0	48.5	1499.9	6.89	1.5	1.236
D	40.1	1152.0	44.7	1431.4	6.28	1.3	1.257
E	40.5	1086.4	48.4	1605.7	5.36	1.1	1.245
F	42.9	1337.7	51.9	1966.0	5.97	1.3	1.274
G	41.0	1242.1	49.4	1481.5	5.10	1.1	1.292

**Table 7:** Summarized mechanical properties of selected FDM-printed NFRPCs.

Natural Fiber	Mechanical Properties	Ref.
Flax and hemp	The tensile strength depended on the fibers’ loading, with higher concentrations of hemp fibers reducing the tensile strength. Other properties, such as flexural strength, decreased with increasing loading of both flax and hemp. The Charpy impact strength increased with fiber loading, with 5 wt. % hemp showing the maximum increase.	[91]
Bamboo	A 26% increase in tensile strength was observed by increasing the infill density between 25 and 100%, whereas this property decreased with increasing layer thickness. Additionally, the stiffness increased between 25 and 40% with increasing infill density. Tensile strength decreased with increasing layer height between 0.2 and 0.3 mm at infill densities between 25 and 100%.	[112]
Flax	Tensile strength and modulus increased by reducing the layer height from 0.6 to 0.2 mm in the longitudinal direction.	[113]
Surface-capped hemp	Tensile, impact, shear, and compressive strengths were 17.56 MPa, 3.43 J/cm^2^, 50 MPa, and 176.69 MPa, respectively.	[114]
Wormwood	Tensile properties (strength, stiffness, and elongation at break) of printed composites were lower compared to neat PLA; however, lower fiber loadings (3 wt. %) showed better performance than composites with 5 wt. % of the fibers. Overall, the observed reduction was attributed to poor interfacial adhesion.	[115]
Basalt	Tensile and flexural strengths increased with the loading of the fibers from 5 to 20 wt. %.	[110]

**Table 8:** Summarized LCA studies on FDM-printed NFRPCs.

NFRPCs System	Selected Impact Categories	Findings	Remarks	Ref.
PLA with 2 and 10% of basalt powder and short hemp fibers	GWP, EP, AP, and CED	Incorporation of 10% basalt fiber and hemp reduced impacts across the evaluated categories. Neat PLA production was the major contributor to GWP 3D printing consumed a high amount of energy	Incorporation of natural fibers or fillers can reduce the environmental impacts of PLA. However, the FDM process remains energy-intensive	[118]
PLA/hemp (straw, hurd, and fiber)	GWP, CED	PLA/hemp fiber filament required the highest CED and contributed more to GWP among the prepared filaments containing hemp hurd and straw	The environmental impacts of NFPRCs in 3D printing are dependent on the filler type and material characteristics.	[119]

## Data Availability

No new data were created or analyzed in this study. Data sharing is not applicable to this article.
